# Sources of information on monkeypox virus infection. A systematic review with meta-analysis

**DOI:** 10.1186/s12889-024-17741-5

**Published:** 2024-01-23

**Authors:** Darwin A. León-Figueroa, Joshuan J. Barboza, Mario J. Valladares-Garrido

**Affiliations:** 1 Facultad de Medicina Humana, Universidad de San Martín de Porres, 15011 Chiclayo, Peru; 2 Unidad de Revisiones Sistemáticas y Meta-análisis, Universidad San Ignacio de Loyola, 15046 Lima, Peru; 3 Universidad Continental, 15046 Lima, Peru; 4 Oficina de Epidemiología, Hospital Regional Lambayeque, 14012 Chiclayo, Peru

**Keywords:** Monkeypox, Knowledge, Attitude, Mpox, Information sources

## Abstract

**Background:**

Monkeypox (Mpox) virus infection is a topic of growing interest today because of its potential public health impact and concern about possible outbreaks. Reliable and up-to-date sources of information that provide accurate data on its transmission, symptoms, prevention, and treatment are essential for understanding and effectively addressing this disease. Therefore, the aim of the present study is to determine the prevalence of sources of information on Mpox virus infection.

**Methods:**

An exhaustive systematic review and meta-analysis was carried out using the information available in the PubMed, Scopus, Web of Science, Embase, and ScienceDirect databases up to August 3, 2023. The data were analyzed using R software version 4.2.3. The quality of the cross-sectional studies that formed part of this review was assessed using the Joanna Briggs Institute Meta-Analysis of Statistics Assessment and Review Instrument (JBI-MAStARI) tool. In addition, a subgroup analysis was performed based on the study populations.

**Results:**

Through electronic searches of five databases, a total of 1833 studies were identified. Twenty-four cross-sectional articles were included, with a total sample of 35,959 participants from 34 countries. The pooled prevalence of each of the included information sources was: social networks reached 59% (95% CI: 50–68%; 29,146 participants; 22 studies; *I*^*2*^ = 100%; *p* < 0.01); the Internet was 61% (95% CI: 44–77%; 14,002 participants; 5 studies; *I*^*2*^ = 100%; *p* < 0.01), radio reached 10% (95% CI: 07–13%; 8917 participants; 4 studies; *I*^*2*^ = 93%; *p* < 0.01), television accounted for 24% (95% CI: 09–43%; 14,896 participants; 8 studies; *I*^*2*^ = 100%; *p* < 0.01), and the combination of radio and television accounted for 45% (95% CI: 31–60%; 4207 participants; 7 studies; *I*^*2*^ = 99%; *p* < 0.01); for newspapers, it was 15% (95% CI: 05–27%; 2841 participants; 6 studies; *I*^*2*^ = 99%; *p* < 0.01), friends and relatives accounted for 19% (95% CI: 12–28%; 28,470 participants; 19 studies; *I*^*2*^ = 100%; *p* < 0.01), the World Health Organization (WHO) accounted for 17% (95% CI: 07–29%; 1656 participants; 3 studies; *I*^*2*^ = 97%; *p* < 0.01), the Centers for Disease Control and Prevention (CDC) accounted for 10% (95% CI: 03–21%; 2378 participants; 3 studies; *I*^*2*^ = 98%; *p* < 0.01), and the combination of WHO and CDC websites accounted for 60% (95% CI: 48–72%; 1828 participants; 4 studies; *I*^*2*^ = 96%; *p* < 0.01), and finally, scientific articles and journals accounted for 24% (95% CI: 16–33%; 16,775 participants; 13 studies; *I*^*2*^ = 99%; *p* < 0.01).

**Conclusion:**

The study suggests that people access a variety of information sources to gain knowledge about Mpox virus infection, with a strong emphasis on online sources such as social networks and the Internet. However, it is important to note that the quality and accuracy of information available from these sources can vary, underscoring the need to promote access to reliable and up-to-date information about this disease to ensure public health.

**Supplementary Information:**

The online version contains supplementary material available at 10.1186/s12889-024-17741-5.

## Introduction

The dissemination of information today has undergone a complete revolution due to the advent of information and communication technologies. In particular, this has transformed the way people obtain and access health-related information [1]. Currently, the global increase in the number of confirmed cases of monkeypox (Mpox) and its rapid spread in various countries around the world have generated a public health emergency of international importance [2].

According to data provided by the Centers for Disease Control and Prevention (CDC) as of December 19, 2023, 92,432 cases of Mpox have been confirmed in 117 countries worldwide [3]. In addition, according to the World Health Organization (WHO), 96.3% of cases (82,158 of 85,286) with available information are male, with a median age of 34 years. Among the various modes of transmission reported, sexual intercourse is the most common, accounting for 82.5% (18,108 of 21,938) of all recorded transmission events [4, 5].

Monkeypox is a zoonotic viral disease caused by a Mpox virus belonging to the genus Orthopoxovirus [6]. Mpox is transmitted mainly through contact with body fluids, skin lesions, or small respiratory droplets of infected animals, either directly or through contaminated objects [7, 8]. The incubation period of Mpox usually ranges from 7 to 21 days, during which non-specific clinical manifestations such as fever, swollen lymph nodes, headache, malaise, and the appearance of skin lesions may occur [9].

With the concern of Mpox disease, people explore, exchange, and obtain health-related information from various sources, such as medical experts, insurance and pharmaceutical entities, close circles of family and friends, media, educational resources, and social networking platforms [10]. However, it is important to have a reliable source of health information to establish a solid understanding of health issues among the population, especially in the current context of the Internet revolution and social media platforms [11]. In addition, approximately 80% of users search for health-related information while on the Internet [12, 13].

Globalization and digital connectivity have drastically altered the way people access medical and health information [14]. Online platforms, social networks, blogs, and news websites have led to a rapid spread of information, but also to the spread of misleading information [15–17].

In this context, understanding the sources of information related to the Mpox virus infection is essential for developing effective public health communication strategies [18, 19]. Since the dissemination of incorrect information can lead to inappropriate responses and decrease trust in health institutions, a thorough assessment of the accuracy and reliability of sources providing data on the Mpox virus becomes crucial [20, 21]. This study seeks to address this knowledge gap by investigating the prevalence of different sources of information about Mpox virus infection.

## Materials and methods

### Protocol and registration

The study procedure has been meticulously documented in the Prospective International Registry of Systematic Reviews (PROSPERO) (**CRD42023456083**), ensuring transparency and completeness in the protocol. During the performance of the systematic review and meta-analysis, the PRISMA (Preferred Reporting Items for Systematic Reviews and Meta-Analyses) checklist standards were followed (Table S1).

### Eligibility criteria

#### Inclusion criteria

All cross-sectional studies that addressed the prevalence of information sources related to Mpox virus infection were included. No limitations in terms of language, time period, or geographical location were applied. However, only those studies that were fully available, provided a detailed description of the sample size, and presented meaningful data regarding any element associated with sources of information on Mpox virus infection were considered.

#### Exclusion criteria

We excluded studies whose research topics did not align with the objectives of our study, as well as those presenting a design that differed from a cross-sectional study approach. Similarly, incomplete papers were discarded, either due to insufficient appropriate data or the absence of relevant information on the desired outcomes. Finally, an attempt was made to establish contact with the main author via e-mail; however, unfortunately, it was not possible to achieve such communication.

### Information sources and search strategy

Extensive searches were conducted in multiple databases, including PubMed, Scopus, Embase, Web of Science, and ScienceDirect. In order to refine and improve the effectiveness of the searches, key terms such as “monkeypox”, “Mpox”, “information sources”, “social media”, “internet sources”, “radio”, “television”, “newspaper”, “friends/family”, “World Health Organization (WHO) website”, “Disease Control and Prevention (CDC) website”, and “research articles/scientific journals” were used. The specific search strategies implemented for each of these databases are detailed in Table S2. The search process was completed on August 3, 2023.

### Study selection

The Rayyan tool was used to efficiently organize and manage the results derived from the search strategy. After eliminating duplicate articles, titles and abstracts were reviewed following predetermined criteria. Subsequently, a thorough analysis of all full-text articles was carried out to assess compliance with the established inclusion criteria. Any discrepancies that arose were addressed through discussions and consultations in collaboration with an expert researcher.

### Main results of the study

This study focused on a fundamental aspect linked to the prevalence of information sources related to the Mpox virus infection.

#### Monkeypox virus information sources

These are various platforms through which participants sought or received information concerning the Mpox virus infection. These sources include social networks, the internet, radio, television, newspapers, family members, and health websites, as well as scientific articles and publications.

### Quality assessment

Two independent investigators used the Joanna Briggs Institute Meta-Analysis of Statistics Assessment and Review Instrument (JBI-MAStARI) tool to assess the quality of the included cross-sectional studies. A third investigator verified the quality assessments of the studies performed. The studies were categorized into high (greater than 7 points), moderate (4 to 6 points), or low (under 4 points) quality levels based on the quality scores assigned (Table S3).

### Data collection process and data items

The data collected from the included studies were recorded in an Excel spreadsheet: name of the lead author, year of publication, country, sample size, study population, sex (male and female), prevalence and number of cases from the sources of information on the Mpox virus infection (social networks, internet, radio, television, newspapers, friends and family, WHO, CDC, scientific articles and publications), type of survey, and date of data collection. To ensure the accuracy and correctness of the information, a researcher conducted a thorough check of the extracted data, eliminating any erroneous data.

### Data analysis

The data extracted from the articles was transferred to a Microsoft Excel spreadsheet. Subsequently, these data were analyzed using R software version 4.2.3. The results obtained were presented in tables and figures.

The estimation of the pooled prevalence of the sources of information on Mpox virus infection was carried out by implementing a random-effects model with inverse variance weighting. To assess the variability between the different studies, the Cochrane Q statistic was used. In addition, quantification of this variability was performed through the *I*^*2*^ index. Values of 25%, 50%, and 75% were considered indicative of low, moderate, and high heterogeneity, respectively.

In order to explore the possible existence of publication bias, a funnel plot was used. Egger’s regression test was also applied to further evaluate this issue. Publication bias was considered to be present when the resulting *p* value was less than 0.05, indicating a possible bias in the results.

In addition, subgroup analyses were performed based on characteristics such as study subjects. The consolidated prevalence of Mpox virus infection information sources was presented using a graphical representation of a forest plot, which incorporated 95% confidence intervals for greater accuracy in the presentation of the results.

## Results

### Study selection

**Fig. 1:**
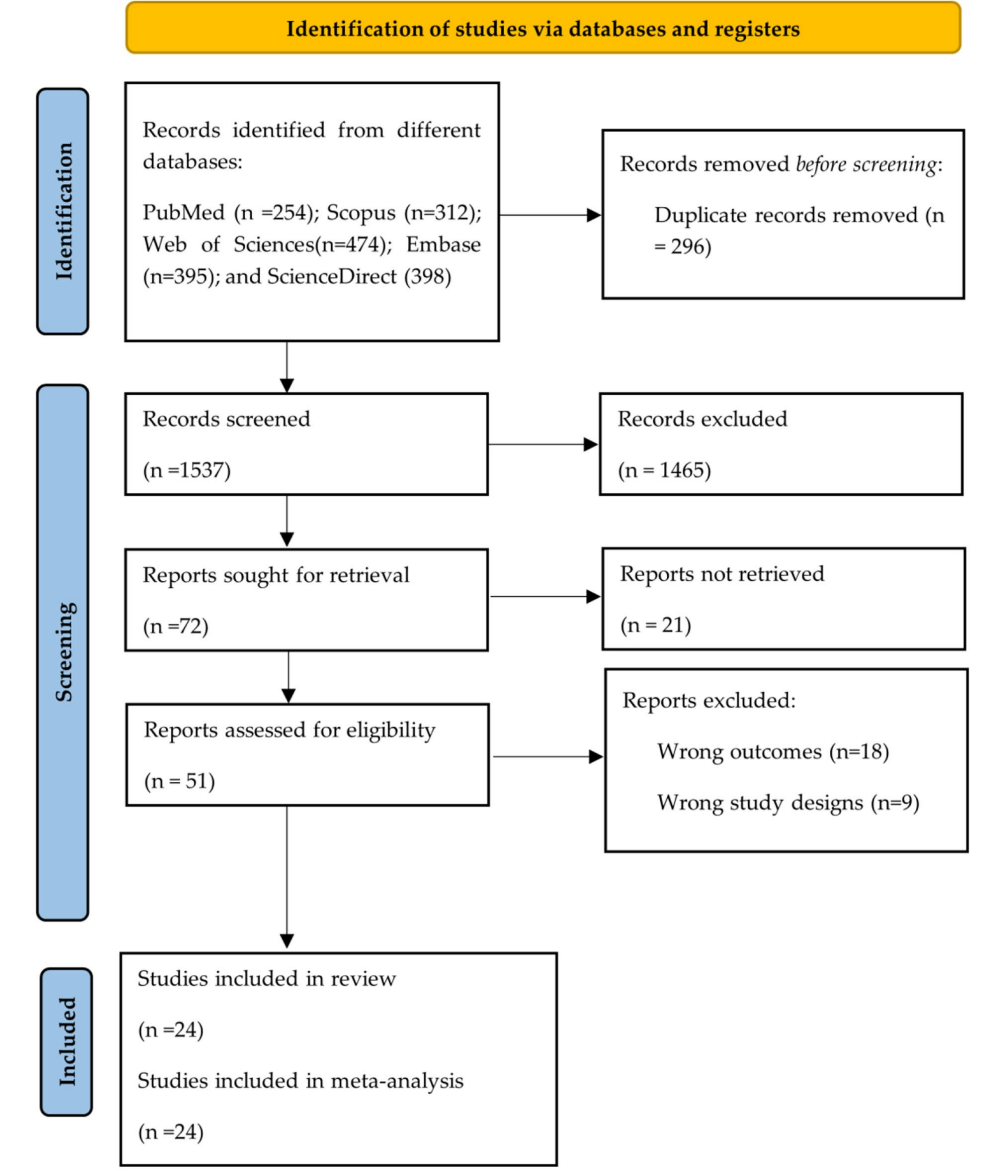
Study selection process based on the PRISMA flowchart

Through electronic searches of five databases, a total of 1,833 studies were identified. After eliminating duplicates (*n* = 296), 1537 studies were evaluated by reviewing titles and abstracts. Of these, 51 were subjected to exhaustive full-text analysis. Finally, 24 studies were included in the final analysis [22–45]. The selection process and the procedure for choosing the articles are described in detail in Fig. 1, following the guidelines established in the PRISMA.

### Characteristics of the included studie

Twenty-four cross-sectional research studies published between 2020 and 2023 were analyzed. A total of 35,959 people participated from 34 countries on four continents: Africa (Algeria, Egypt, Ethiopia, Morocco, Nigeria, Senegal, South Africa, Sudan, and Tanzania), Asia (Bahrain, Bangladesh, Georgia, India, Iraq, Jordan, Lebanon, Malaysia, Pakistan, Palestine, Saudi Arabia, Syria, the United Arab Emirates, Yemen, the Philippines, China, Turkey, and Indonesia), America (Peru and Brazil), and Europa (Greece, Poland, Romania, the United Kingdom, and the Czech Republic). Of these participants, 56.15% (*n* = 20,190) were men, while 43.72% (*n* = 15,723) were women [22–45]. The questionnaires used for data collection were applied exclusively through online surveys, specifically designed for different population groups, ranging from the general population to health professionals and members of the lesbian, gay, bisexual, transgender, intersex, queer, and more (LGBTIQ+) community. Table 1 summarizes the particularities of the research considered [22–45].

**Table 1:** Characteristics of the included studies on the sources of information on monkeypox virus infection

Authors	Year	Study design	Country	Sample size (n)	Study population	Sex		Social media	Internet sources	Radio	TV	Newspaper	Friends/Family	WHO website	CDC website	Research articles/ Scientific journals	Survey Type	Data Collection Date
						M	F											
Abd ElHafeez S, et al. [22]	2023	Cross sectional	27 countries	11,919	Medical Students	5432	6487	8788 (73.7%)	NR	NR	NR	NR	Friends: 5183 (43.5%) Family: 4500 (37.8%)	NR	NR	4451 (37.3%)	Online survey	September 1 to December 15, 2022
Berdida DJE. [23]	2023	Cross sectional	Philippines	575	General population	179	396	452 (78.61%)	NR	87 (15.13%)			36 (6.26%)	NR	NR	NR	Online survey	July - September 2022
Awoyomi OJ, et al. [24]	2023	Cross sectional	Nigeria	1544	General population	938	606	479 (31%)	NR	185 (12%)	448 (29%)		185 (12%)	NR	247 (16%)	NR	Online survey	24 July − 12 August 2022
Gonzales-Zamora JA, et al. [25]	2023	Cross sectional	Peru	463	Healthcare Workers	269	194	206 (44.4%)	NR	232 (50.0%)		96 (20.7%)	145 (31.3)	335 (72.2%)		180 (38.8%)	Online survey	10 August − 4 September 2022
Abu-Farha RK, et al. [26]	2023	Cross sectional	Jordan	1195	General population	524	671	1019 (85.2%)	NR	741 (62.1%)		NR	557 (46.6%)	NR	NR	NR	Online survey	September - October 2022
Araoz-Salinas JM, et al. [27]	2023	Cross sectional	Peru	373	LGBTI community	317*	23	242 (64.7%)	NR	246 (65.9%)		65 (17.5%)	104 (28%)	175 (46.9%)		27 (7.3%)	Online survey	1 November 2022–17 January 2023
Sobaikhi NH, et al. [28]	2023	Cross sectional	Saudi Arabia	398	Healthcare Workers	246	152	274 (68.8%)	NR	NR	NR	NR	NR	NR		NR	Online survey	4 November − 8 December 2022
Alhasan K, et al. [29]	2023	Cross sectional	Saudi Arabia	199	Healthcare Workers	74	125	96 (48.2%)	NR	NR	NR	NR	NR	136 (68.3%)		61 (30.7%)	Online survey	15 August − 5 September 2022
Torres TS, et al. [30]	2023	Cross sectional	Brazil	6236	LGBTI community	6236*	0	NR	4793 (76.9%)	853 (13.7%)	4519 (72.5%)	NR	NR	NR	NR	NR	Online survey	October - November 2022
Shafei AM, et al. [31]	2023	Cross sectional	Saudi Arabia	315	Healthcare Workers	208	107	152 (48.25%)	NR	12 (3.81%)	33 (10.48%)	NR	NR	NR	NR	126 (40%)	Online survey	1 March − 1 May 2023
Al-Mustapha AI, et al. [32]	2023	Cross sectional	Nigeria	822	General population	472	342	209 (28.6%)	244 (33.4%)	94 (12.9%)	187 (25.6%)	49 (6.7%)	54 (7.4%)	73 (10%)	NR	NR	Online survey	16–29 August 2022
Ibrahim AM, et al. [33]	2023	Cross sectional	Saudi Arabia	960	Medical Students	422	538	557 (58%)	NR	192 (20%)		NR	58 (6%)	NR	NR	NR	Online survey	November 2022 - January 2023
Fu L, et al. [34]	2023	Cross sectional	China	577	LGBTI community	577*	0	NR	461 (79.9%)	NR	166 (28.8%)		25 (4.3%)	NR	NR	NR	Online survey	10 August − 9 September 2022
Jamaleddine Y, et al. [35]	2023	Cross sectional	Lebanon	493	General population	119	374	261 (52.9%)	152 (30.8)	NR	111 (22.5%)	NR	80 (16.2%)	134 (27.2%)	82 (16.6%)	NR	Online survey	6–20 September 2022
Ahmed SK, et al. [36]	2023	Cross sectional	Iraq	510	General population	277	233	316 (62%)	NR	NR	56 (11%)		46 (9%)	NR	NR	NR	Online survey	27–30 July 2022
Swed S, et al. [37]	2023	Cross sectional	Arabic regions	5874	Healthcare Workers	2455	3419	5280 (89.9%)	4858 (82.7%)	NR	2218 (37.8%)	NR	3116 (53.0%)	NR	NR	NR	Online survey	6–25 June 2022
Elkhwesky Z, et al. [38]	2023	Cross sectional	Egypt	453	Hotel employees	363	90	351 (77.5%)	NR	220 (48.6%)		NR	212 (46.8%)	NR	NR	141 (31.1%)	Online survey	September 2022
Youssef D, et al. [39]	2023	Cross sectional	Lebanon	793	General population	231	562	463 (58.39%)	NR	545 (68.73%)			350 (44.14%)	422 (53.22%)		420 (52.96%)	Online survey	August 2022
Sahin TK, et al. [40]	2022	Cross sectional	Turkey	283	Physicians	117	166	147 (51.9%)	NR	79 (27.9%)		NR	45 (15.9%)	NR	NR	138 (48.8%)	Online survey	20 August–2 September 2022
Alshahrani NZ, et al. [41]	2022	Cross sectional	Saudi Arabia	314	Medical Students	131	183	195 (62.1%)	NR	NR	97 (30.8%)	NR	13 (4.2%)	NR	NR	46 (14.7%)	Online survey	24 May − 20 July 2022
Alshahrani NZ, et al. [42]	2022	Cross sectional	Saudi Arabia	480	General population	198	282	360 (75.0%)	NR	219 (45.6%)		NR	75 (15.6%)	NR	NR	42 (8.8%)	Online survey	25 May − 15 July 2022
Riad A, et al. [43]	2022	Cross sectional	Czech Republic	341	Healthcare Workers	33	303	88 (25.8%)	NR	NR	NR	162 (47.5%)	NR	55 (16.1%)	5 (1.5%)	19 (5.6%)	Online survey	September 2022
Kaur A, et al. [44]	2022	Cross sectional	India	410	Dental professionals	232	178	113 (42.2%)	NR	NR	44 (16.4%)	27 (10%)	43 (16.1%)	NR	NR	41 (15.3%)	Online survey	June 2022
Harapan H, et al. [45]	2020	Cross sectional	Indonesia	432	Physicians	140	292	318 (73.6%)	NR	NR	12 (2.8%)	15 (3.5%)	43 (10%)	NR	NR	41 (9.6%)	Online survey	25 May − 25 July 2019

M/F: Male/Female; NR: Not reported; TV: Television; World Health Organization (WHO) website, Centers for Disease Control and Prevention (CDC) website, *MSM: men who have sex with men; and the lesbian, gay, bisexual, transgender, and intersex (LGBTI) community

### Quality of the included studies and publication bias

The included cross-sectional studies were distinguished by their moderate level of quality, as determined by the JBI-MAStARI instrument (Table S3) [23–45]. Publication bias was examined in articles that mentioned sources of information from social networks, friends, or family, as well as articles or journals, since more than 10 of them referred to these sources of information (Figure S1). The application of Egger’s test to assess publication bias in studies that addressed social networks as a source of information on Mpox yielded a value of *p* = 0.0128 (t = -2.73, df = 20). This result led to the rejection of the null hypothesis of symmetry, possibly indicating the presence of a publication bias (Figure S1, a). Egger’s test, used to evaluate publication bias in articles that considered friends or relatives as a source of information on Mpox, yielded a value of *p* = 0.0004 (t = -4.38, df = 17). This result leads to the rejection of the null hypothesis of symmetry, possibly indicating the presence of a publication bias (Figure S1, b). Egger’s test, used to assess publication bias in articles that considered other publications or journals as a source of information on Mpox, revealed a value of *p* = 0.0563 (t = -2.13, df = 11). This led to the acceptance of the null hypothesis of symmetry, possibly suggesting the absence of publication bias (Figure S1, c).

### Sources of information on Mpox virus

The combined prevalence of information sources about the Mpox virus on social networks reached 59% (95% CI 50–68%; 29,146 participants; 22 studies; *I*^*2*^ = 100%; *p* < 0.01) (Figure S2) [22–29, 31–33, 35–45]. On the other hand, access via the internet accounted for 61% (95% CI: 44–77%; 14,002 participants; 5 studies; *I*^*2*^ = 100%; *p* < 0.01) (Figure S3) [30, 32, 34, 35, 37]. As for radio, a level of 10% (95% CI: 07–13%; 8917 participants; 4 studies; *I*^*2*^ = 93%; *p* < 0.01) was observed (Figure S4) [24, 30–32], while television accounted for 24% (95% CI: 09–43%; 14,896 participants; 8 studies; *I*^*2*^ = 100%; *p* < 0.01) (Figure S5) [30–32, 35, 37, 42, 44, 45]. The combination of radio and television accounted for 45% (95% CI: 31–60%; 4207 participants; 7 studies; *I*^*2*^ = 99%; *p* < 0.01) (Figure S6) [25–27, 33, 38, 40, 41]. In the case of newspapers, it accounted for 15% (95% CI: 05–27%; 2841 participants; 6 studies; *I*^*2*^ = 99%; *p* < 0.01) (Figure S7) [25, 27, 32, 43–45].

Friends and relatives accounted for 19% (95% CI 12–28%; 28,470 participants; 19 studies; *I*^*2*^ = 100%; *p* < 0.01) (Figure S8) [22–27, 32–42, 44, 45]. The WHO websites accounted for 17% (95% CI 07–29%; 1656 participants; 3 studies; *I*^*2*^ = 97%; *p* < 0.01) (Figure S9) [32, 35, 43], whereas CDC websites contributed 10% (95% CI 03–21%; 2378 participants; 3 studies; *I*^*2*^ = 98%; *p* < 0.01) (Figure S10) [24, 35, 43]. The combination of WHO and CDC websites comprised 60% (95% CI 48–72%; 1828 participants; 4 studies; *I*^*2*^ = 96%; *p* < 0.01) (Figure S11) [25, 27, 29, 39]. Finally, scientific articles and journals accounted for 24% (95% CI 16–33%; 16,775 participants; 13 studies; *I*^*2*^ = 99%; *p* < 0.01) (Figure S12) [25, 27, 29, 31, 38–45].

### Subgroup analysis by study population

The prevalence of information sources from social networks regarding Mpox virus among study participants stood at 61% (95% CI: 45–77%; 6865 participants; 9 studies; *I*^*2*^ = 99%; *p* < 0.01) [23, 24, 26, 32, 35, 36, 38, 39, 41] in the general population, and 57% (95% CI: 45–68%; 21,908 participants; 12 studies; *I*^*2*^ = 100%; *p* < 0.01) [22, 25, 28, 29, 31, 33, 37, 40, 42–45] in the health care worker group (Figure S13).

The prevalence of information sources from television about the Mpox virus among study participants was established at 23% (95% CI: 20–25%; 1315 participants; 2 studies; *I*^*2*^ = 0%; *p* = 0.93) in the general population [32, 35], whereas in the group of health care workers it was 16% (95% CI: 04–35%; 7345 participants; 5 studies; *I*^*2*^ = 99%; *p* < 0.01) [31, 37, 42, 44, 45] (Figure S14).

The prevalence of information sources from newspapers regarding the Mpox virus among study participants was 17% (95% CI: 03–38%; 1646 participants; 4 studies; *I*^*2*^ = 99%; *p* < 0.01) in the health worker group (Figure S15) [25, 43–45].

The prevalence of information sources originating from friends and family regarding the Mpox virus among study participants stood at 20% (95% CI: 10–33%; 6865 participants; 9 studies; *I*^*2*^ = 99%; *p* < 0.01) in the general population [23, 24, 26, 32, 35, 36, 38, 39, 42], while in health care workers it was 19% (95% CI: 10–32%; 20,655 participants; 8 studies; *I*^*2*^ = 100%; *p* < 0.01) [22, 25, 33, 37, 40, 41, 44, 45]. In the LGBTIQ + community, this prevalence stood at 14% (95% CI: 00–44%; 950 participants; 2 studies; *I*^*2*^ = 99%; *p* < 0.01) [27, 34] (Figure S16).

The prevalence of information sources from scientific articles and scientific journals about the Mpox virus among study participants was set at 29% (95% CI: 07–59%; 1726 participants; 3 studies; *I*^*2*^ = 99%; *p* < 0.01) in the general population [38, 39, 42], whereas in health care workers it was 24% (95% CI: 15–35%; 14,676 participants; 9 studies; *I*^*2*^ = 99%; *p* < 0.01) [25, 29, 31, 40, 42–45] (Figure S17).

## Discussion

The recent outbreak of the Mpox, confirmed by the WHO, highlights the need to analyze the sources of information related to the Mpox virus and to understand which information sources are most commonly used by both the general population and health professionals. At present, knowledge about Mpox continues to develop constantly, making it essential to stay informed through reliable sources of information. The present systematic review determined the prevalence of different sources of information about Mpox virus infection. The main sources of information were the Internet, social networks, and the joint use of radio and television.

The use of the Internet as a source of information about the Mpox virus represented a prevalence of 61%. According to a study conducted by Swed S. et al., 82.7% of health professionals used the Internet for this purpose [37]. Another study conducted by Fu L. et al. found that 79.9% of men who have sex with men used the Internet as a primary source of information about the Mpox virus [34]. In addition, research by Torres TS et al. found that sexual and gender minorities had a high rate of Internet use for information about the Mpox virus, with a prevalence of 76.9% [30]. These findings demonstrate the growing reach of the Internet in society and its remarkable importance as a communication platform and source of information. This process democratizes the availability of information about the Mpox virus, but at the same time creates challenges regarding the veracity of data and the urgent importance of promoting digital literacy. In addition, the preponderance of the Internet as a source of information points to a shift in communication channels, which could have significant implications for how crucial health information is disseminated to the general population. It also highlights the need to recognize inequalities in Internet access among various demographic segments.

Social media reached a prevalence of 59% as a source of information about the Mpox virus. When analyzing these data by subgroups, it was found that both the general population and health care workers obtained a prevalence of 61% and 57%, respectively, when using social networks as a source of information.

According to various studies conducted in different regions of the world, similar results have been observed regarding the use of social networks as a source of information about the Mpox virus. For example, in the Philippines, Berdida DJE et al. reported that 78.61% of the population resorted to social networks for this purpose [23]. A study conducted in 27 countries with a sample of 11,919 participants and conducted by Abd ElHafeez S. et al. revealed that 73.7% of participants used social networks as a source of information about Mpox [22]. In Jordan, Abu-Farha RK and his team reported an even higher prevalence, reaching 85.2% of the population [26]. Similarly, a study conducted in the LGBTIQ + community in Peru by Araoz-Salinas JM et al. documented that 64.7% of this community used social networks as a source of information about the Mpox virus [27]. In Saudi Arabia, Sobaikhi NH et al. found that 68.8% of health care workers relied on social networks as their main source of information about this virus [28]. Finally, a study by Swed S. and colleagues, which included 5874 medical students and clinicians from 17 Arab countries, reported that an impressive 89.9% of participants relied on social networks as their primary source of information about the Mpox virus [37].

These results highlight that social media play an important role in the spread of information about the Mpox virus, underscoring the strong influence of these platforms in the dissemination of public health-related data. Still, this also raises concerns about the reliability of the information, given that social networks can serve as both trusted sources and vehicles of misinformation. Therefore, it is crucial that health authorities and communicators promote accurate information in these media.

Radio was established as a source of information with a prevalence of 10%; in contrast, television occupied a more prominent place, representing 24%. When both media—radio and television—are combined, their combined prevalence reaches 45%. However, when examining these data by subgroups, it is evident that the general population and health care workers show a different prevalence. In the case of the general population, television as a source of information reached a prevalence of 23%, while health workers showed a prevalence of 16% for this medium.

Similar results have been found in several studies on the use of radio and television as sources of information about the Mpox virus in different regions of the world. For example, Awoyomi OJ et al. in Nigeria reported a prevalence of 12% in the use of radio as a source of information about the Mpox virus [24]. In Brazil, Torres TS and his team reported that 13.7% of the population used radio for information about the virus, while 72.5% preferred television as a source of information [30]. In another study also conducted in Nigeria by Al-Mustapha AI and colleagues, prevalences of 12.9% and 25.6% were recorded in the use of radio and television, respectively, as sources of information about the Mpox virus [30]. In addition, studies conducted by Ibrahim AM and colleagues, as well as Araoz-Salinas JM and his team, found prevalence rates of 20% and 65.9% in the joint use of radio and television as sources of information about the Mpox virus [27, 33].

These results suggest that television and radio are significant sources of information for the majority of the population, but the prevalence of television varies between the general population and health care workers. This could be due to differences in access, preferences, and perceived credibility of information sources in these groups. Health professionals tend to seek highly specialized and up-to-date information in their fields, which reduces their reliance on television compared to the general public. This is due to their preference for information related to medical and scientific research, which explains why they use television less, while the general population seeks a wider variety of content. In addition, health care workers tend to be more critical of the credibility of information sources, leading them to have less trust in television compared to the general population, which is often more influenced by television as a form of entertainment.

In the case of newspapers, it was found that they represented a source of information on the Mpox virus with a prevalence of 15%. However, when analyzing the data by subgroups, it was observed that health workers showed a higher prevalence of 17% when using newspapers as a source of information.

In studies conducted in 2022, similar results were found in different geographic and population contexts regarding the use of newspapers as a source of information about the Mpox virus. In Peru, Gonzales-Zamora JA and colleagues reported that 20.7% of Peruvian physicians resorted to newspapers as an information source during the 2022 outbreak [25]. In the same period, Araoz-Salinas JM et al. reported that 17.5% of the LGBTIQ + community also used newspapers as an information resource about the Mpox virus [27]. On the other hand, Riad A. et al. conducted a study among Czech healthcare workers and found that 47.5% of them used digital news newspapers as a source of information about the Mpox virus [43].

The results of this study highlight the continued relevance of newspapers as a source of information on Mpox, especially among health workers. Data segmentation is crucial for understanding the informational preferences and needs of different demographic groups, such as health workers, who show a greater reliance on newspapers as a source of information. This has significant implications for public health communication, as it suggests the possibility of collaboration with print media to ensure the accuracy and timeliness of information targeted at this group. In addition, it highlights the importance of critically evaluating the quality of information in newspapers, as their prevalence as a source does not guarantee its reliability, underscoring the need to promote education on the critical evaluation of information sources, including print media.

Friends and family members presented a prevalence of 19% as a source of information about the Mpox virus. However, when performing an analysis by subgroups, it was observed that the general population, health workers, and the LGBT community reported prevalences of 20%, 19%, and 14%, respectively, when using friends and family as their main source of information.

Based on the data provided, several studies have been conducted on the prevalence of using friends and family as a source of information about the Mpox virus in different regions. According to a study conducted by Abd ElHafeez S. et al., 43.5% of the participants consulted their friends as a source of information about Mpox, while 37.8% preferred to obtain information from their relatives [22]. Awoyomi OJ and colleagues reported a prevalence of 12% in Nigeria [24]. In Peru, Gonzales-Zamora JA and team found a prevalence of 31.3% among Peruvian physicians during the 2022 outbreak [25]. Abu-Farha RK et al. reported a prevalence of 42.6% in Jordan [26]. In addition, Araoz-Salinas JM and colleagues found a prevalence of 28% among the LGBTIQ + community in Peru during the same outbreak [27]. A larger study involving 5874 medical students and clinicians from 17 Arab countries, led by Swed S. and his team, revealed that 53% of participants relied primarily on friends and family as a source of information about the Mpox virus [37].

The results reveal that in the general population, there is a greater tendency to turn to friends and family as a source of information about the Mpox virus. This preference could be attributed to the high degree of trust that people tend to place in their close circle, possibly due to the absence of specialized medical training in most cases.

The websites of the WHO and the CDC accounted for 17% and 10%, respectively, as a source of information on the Mpox virus. The combination of both websites, that is, the joint use of WHO and CDC, accounted for a total of 60% of the information sources used.

According to available data, similar results have been reported in several countries regarding the use of the WHO and the CDC as sources of information on the Mpox virus. In Nigeria, Al-Mustapha AI and colleagues found a 10% prevalence in the use of WHO as a source of information on the Mpox virus [32]. In Lebanon, Jamaleddine Y. et al. reported prevalences of 27.2% and 16.6% in the use of WHO and CDC, respectively, as sources of information on the Mpox virus [35]. In the Czech Republic, Riad A. et al. reported a prevalence of 16.1% in the use of WHO as a source of information on the Mpox virus [43]. In Nigeria, Awoyomi OJ et al. reported a 16% prevalence of using the CDC as a source of information on the Mpox virus [24]. In Peru, during the 2022 outbreak, Gonzales-Zamora JA et al. reported a 72.2% prevalence of joint use of WHO and CDC as sources of information on the Mpox virus [25]. In another study conducted in the Lebanese population by Youssef D. et al., a prevalence of 53.22% was found for the joint use of WHO and CDC as a source of information on the Mpox virus [39].

The results of the study indicate that both WHO and CDC are reliable sources of information on the Mpox virus, and the combination of their websites is widely used. This suggests that people may feel more confident getting information from sources supported by public health experts. This highlights the importance of effective communication by public health authorities.

Finally, scientific articles and journals as a source of information on the Mpox virus accounted for 24%. When analyzing subgroups, it was observed that the general population and health care workers presented a prevalence of 29% and 24%, respectively, when using scientific articles and journals as a source of information. According to the information provided, similar results have been reported in several studies on the use of scientific articles and journals as a source of information on the Mpox virus in different regions and population groups.

In Peru, Gonzales-Zamora JA, et al. found a prevalence of 38.8% among Peruvian physicians during the 2022 outbreak [25]. In addition, Araoz-Salinas JM et al. documented a prevalence of 7.3% in the LGBTIQ + community in Peru [27]. In Saudi Arabia, Alhasan K. et al. reported a prevalence of 30.7% among healthcare workers [29], while Shafei AM. et al. reported a prevalence of 40% in the same group [31]. Sahin TK reported a prevalence of 48.8% among health care workers in terms of the use of scientific articles and journals as a source of information on the Mpox virus [40]. Finally, in a study conducted in the Lebanese population by Youssef D. et al., a prevalence of 52.96% was found for the use of scientific articles and journals as a source of information on the Mpox virus [39].

The results suggest that the preference for using scientific articles and journals as a source of information about the Mpox virus in the general population may reflect a desire for accurate, evidence-based information. This is encouraging, as it indicates an interest in acquiring solid knowledge about the disease rather than relying solely on less reliable sources such as rumors or social networks. On the other hand, the choice of this source by health care workers could be due to their familiarity with medical terminology and research methodology, which allows them to better understand and apply information from scientific publications in their professional practice.

In this study, it has been observed that variations among different information sources and populations can be attributed to a complex interaction of factors, including cultural, socioeconomic, and professional differences. In the case of healthcare professionals, it has been identified that they use television less frequently than the rest of the population. This tendency can be attributed to their demanding work schedules, which often limit their free time for leisure activities. In addition, their educational background and specialized training may make them more critical when selecting information sources. They prefer to access scientific studies and specialized literature rather than rely on mass media [46].

The diversity in healthcare professionals’ engagement with the scientific literature is influenced by a number of factors, such as their specific professions, geographic regions, and other contextual aspects. Differences in the availability of resources, access to continuing education, and job expectations can play an important role in determining health professionals’ commitment to staying current in the scientific field. In addition, cultural and structural variations in health systems, as well as differences in the needs of the communities they serve, may also contribute to disparities in engagement with the scientific literature [47].

A fundamental pillar when analyzing the most frequently consulted sources of information on Mpox lies in the fact that misinformation in the health field can have potentially devastating consequences. It can lead to the proliferation of conspiracy theories and false treatments, sow unnecessary fear, discourage vaccination, and create an environment conducive to epidemic outbreaks. In addition, misinformation can undermine confidence in public health authorities and vaccination programs, making it difficult to respond effectively to potential outbreaks and increasing the risk of the disease spreading unchecked [48, 49].

To address this problem, it is crucial to ensure the accurate dissemination of information about the Mpox virus infection. It is essential that public health authorities and policymakers implement comprehensive communication strategies. First, it is essential to establish clear and accessible communication channels, such as official websites and verified social networks, where up-to-date and reliable information on Mpox can be found. These resources should be easy to understand for the general public and should include details on symptoms, prevention methods, and treatment. In addition, it is crucial to collaborate with social media platforms and search engines to improve the visibility of reliable resources and verify the authenticity of information shared online. Awareness campaigns on social media and the promotion of educational content produced by health experts can help counter misinformation and reach a wider audience [20, 50, 51].

In addition, authorities should invest in educational programs aimed at increasing digital literacy among the population, teaching people to discern between reliable and false information online. This may include creating educational materials, workshops, and seminars to encourage critical thinking and the evaluation of information on the Internet. It is also important to collaborate with influencers and public figures to disseminate accurate and reliable messages about Mpox, leveraging their reach on social media to reach diverse audiences. By working together with communication and technology experts, authorities can ensure that accurate information about Mpox virus infection reaches the population effectively, helping to prevent the spread of the disease and protect public health [20, 50, 51].

The study had some limitations. First, the reports related to sources of information on Mpox were not addressed in a stand-alone manner but were embedded in research designed to assess knowledge, attitudes, and practices in relation to Mpox. Second, in our search strategy, the Chinese public approach was not considered a source of information on the Mpox virus from the Chinese CDC, which could have provided valuable data over the past few years. In addition, it is crucial to recognize the possible presence of bias in the included observational studies, whether in sample selection, data collection methodology, or even in the subjective interpretations of the investigators. Despite this, these studies managed to address these challenges through the application of advanced statistical techniques, rigorous methodology, and careful interpretation of their results, comparing them with previously published studies. It is relevant to keep in mind that the studies incorporated in the meta-analysis were conducted in different populations and geographic regions. This study represents the first systematic review and meta-analysis that evaluates the various sources of information used by the general population, health personnel, and the LGBTIQ + community about the Mpox virus infection. In addition, it is important to highlight that a rigorous methodology was implemented, which was carried out following the criteria established by PRISMA. In addition, it is worth mentioning that the selection of the studies was carried out independently by at least two authors, which strengthens the reliability of the results obtained.

## Conclusions

The study suggests that people access a variety of information sources to gain knowledge about the Mpox virus infection, with a strong emphasis on online sources such as social networks and the Internet. However, it is important to note that the quality and accuracy of information available from these sources can vary, underscoring the need to promote access to reliable and up-to-date information about this disease to ensure public health.

### Electronic supplementary material

Below is the link to the electronic supplementary material.

Supplementary Material 1

## Data Availability

All data generated or analyzed during this study are included in this published article.
