# Supplementary material for: Sources of information on monkeypox virus infection. A systematic review with meta-analysis
Source: BMC Public Health. 2024 Jan 23;24:276. doi: 10.1186/s12889-024-17741-5 (PMC10807226; doi:10.1186/s12889-024-17741-5)
Supplement: Supplementary file 1 — Supplementary Material 1 [file 12889_2024_17741_MOESM1_ESM.docx]

Systematic Review

Sources of information on monkeypox virus infection. A systematic review with meta-analysis.

Darwin A. León-Figueroa ^1^, Joshuan J. Barboza ^2^, Mario J. Valladares-Garrido ^3,4,^ *

^1^ Facultad de Medicina Humana, Universidad de San Martín de Porres, Chiclayo 15011, Peru; [darwin_leon@usmp.pe](mailto:darwin_leon@usmp.pe)

^2^ Unidad de Revisiones Sistemáticas y Meta-análisis, Universidad San Ignacio de Loyola, Lima 15046, Peru; [jbarbozameca@gmail.com](mailto:jbarbozameca@gmail.com)

^3^ Universidad Continental, Lima 15046, Peru; [mvalladares@continental.edu.pe](mailto:mvalladares@continental.edu.pe)

^4^ Oficina de Epidemiología, Hospital Regional Lambayeque, Chiclayo 14012, Peru

***** **Correspondence**: Correspondence: [mvalladares@continental.edu.pe](mailto:mvalladares@continental.edu.pe) ; Tel.: +51 944 655 396.

**Supplementary material**

- **Table S1. PRISMA Checklist** (PRISMA 2020 Main Checklist and PRIMSA Abstract Checklist)
- **Table S2.** The adjusted search terms as per searched electronic databases.
- **Table S3.** Quality of the included studies
- **Figure S1**: Funnel plot and Egger's test illustrate the publication bias of the included studies.
- **Figure S2.** Forest plot showing the prevalence of social networks as a source of information about monkeypox virus infection.
- **Figure S3.** Forest plot showing the prevalence of the Internet as a source of information on monkeypox virus infection.
- **Figure S4.** Forest plot showing the prevalence of Radio as a source of information about monkeypox virus infection.
- **Figure S5.** Forest plot showing the prevalence of Television as a source of information about monkeypox virus infection.
- **Figure S6.** Forest plot showing the prevalence of Radio/Television as a source of information about monkeypox virus infection.
- **Figure S7.** Forest plot showing the prevalence of newspaper as a source of information on monkeypox virus infection.
- **Figure S8.** Forest plot showing the prevalence of Friends/Relatives as a source of information about monkeypox virus infection.
- **Figure S9.** Forest plot showing the prevalence of the WHO website as a source of information on monkeypox virus infection.
- **Figure S10.** Forest plot showing the prevalence of the CDC website as a source of information on monkeypox virus infection.
- **Figure S11.** Forest plot showing the prevalence of WHO and CDC websites as a source of information on monkeypox virus infection.
- **Figure S12.** Forest plot showing the prevalence of research articles/scientific journals as a source of information on monkeypox virus infection.
- **Figure S13.** Graphical representation of the prevalence of information sources in social networks about monkeypox virus among study participants.
- **Figure S14.** Graphical representation of the prevalence of information sources in television about monkeypox virus among study participants.
- **Figure S15.** Graphical representation of the prevalence of information sources in newspapers about monkeypox virus among study participants.
- **Figure S16.** Graphical representation of the prevalence of information sources in Friends/Family about monkeypox virus among study participants.
- **Figure S17**. Graphical representation of the prevalence of information sources in Research articles/ Scientific journals about monkeypox virus among study participants.
- **References**

**Table S1. PRISMA Checklist** (PRISMA 2020 Main Checklist and PRIMSA Abstract Checklist)

# PRISMA 2020 Main Checklist

| **Topic** | **No.** | **Item** | **Location where item is reported** |
| --- | --- | --- | --- |
| **TITLE** |  |  |  |
| **Title** | 1 | Identify the report as a systematic review. | 1 |
| **ABSTRACT** |  |  |  |
| **Abstract** | 2 | See the PRISMA 2020 for Abstracts checklist |  |
| **INTRODUCTION** |  |  |  |
| **Rationale** | 3 | Describe the rationale for the review in the context of existing knowledge. | 3 |
| **Objectives** | 4 | Provide an explicit statement of the objective(s) or question(s) the review addresses. | 4 |
| **METHODS** |  |  |  |
| **Eligibility criteria** | 5 | Specify the inclusion and exclusion criteria for the review and how studies were grouped for the syntheses. | 4 |
| **Information sources** | 6 | Specify all databases, registers, websites, organisations, reference lists and other sources searched or consulted to identify studies. Specify the date when each source was last searched or consulted. | 4 |
| **Search strategy** | 7 | Present the full search strategies for all databases, registers and websites, including any filters and limits used. | 5 |
| **Selection process** | 8 | Specify the methods used to decide whether a study met the inclusion criteria of the review, including how many reviewers screened each record and each report retrieved, whether they worked independently, and if applicable, details of automation tools used in the process. | 5 |
| **Data collection process** | 9 | Specify the methods used to collect data from reports, including how many reviewers collected data from each report, whether they worked independently, any processes for obtaining or confirming data from study investigators, and if applicable, details of automation tools used in the process. | 5 |
| **Data items** | 10a | List and define all outcomes for which data were sought. Specify whether all results that were compatible with each outcome domain in each study were sought (e.g. for all measures, time points, analyses), and if not, the methods used to decide which results to collect. | 5-6 |
|  | 10b | List and define all other variables for which data were sought (e.g. participant and intervention characteristics, funding sources). Describe any assumptions made about any missing or unclear information. | 6 |
| **Study risk of bias assessment** | 11 | Specify the methods used to assess risk of bias in the included studies, including details of the tool(s) used, how many reviewers assessed each study and whether they worked independently, and if applicable, details of automation tools used in the process. | 6 |
| **Effect measures** | 12 | Specify for each outcome the effect measure(s) (e.g. risk ratio, mean difference) used in the synthesis or presentation of results. | 6 |
| **Synthesis methods** | 13a | Describe the processes used to decide which studies were eligible for each synthesis (e.g. tabulating the study intervention characteristics and comparing against the planned groups for each synthesis (item 5)). | 6 |
|  | 13b | Describe any methods required to prepare the data for presentation or synthesis, such as handling of missing summary statistics, or data conversions. | 6 |
|  | 13c | Describe any methods used to tabulate or visually display results of individual studies and syntheses. | 6 |
|  | 13d | Describe any methods used to synthesize results and provide a rationale for the choice(s). If meta-analysis was performed, describe the model(s), method(s) to identify the presence and extent of statistical heterogeneity, and software package(s) used. | 6 |
|  | 13e | Describe any methods used to explore possible causes of heterogeneity among study results (e.g. subgroup analysis, meta-regression). | 6 |
|  | 13f | Describe any sensitivity analyses conducted to assess robustness of the synthesized results. | 6 |
| **Reporting bias assessment** | 14 | Describe any methods used to assess risk of bias due to missing results in a synthesis (arising from reporting biases). | 6 |
| **Certainty assessment** | 15 | Describe any methods used to assess certainty (or confidence) in the body of evidence for an outcome. | 6 |
| **RESULTS** |  |  |  |
| **Study selection** | 16a | Describe the results of the search and selection process, from the number of records identified in the search to the number of studies included in the review, ideally using a flow diagram. | 7 |
|  | 16b | Cite studies that might appear to meet the inclusion criteria, but which were excluded, and explain why they were excluded. | 7 |
| **Study characteristics** | 17 | Cite each included study and present its characteristics. | 7 |
| **Risk of bias in studies** | 18 | Present assessments of risk of bias for each included study. | 7-8 |
| **Results of individual studies** | 19 | For all outcomes, present, for each study: (a) summary statistics for each group (where appropriate) and (b) an effect estimate and its precision (e.g. confidence/credible interval), ideally using structured tables or plots. | 7-8 |
| **Results of syntheses** | 20a | For each synthesis, briefly summarise the characteristics and risk of bias among contributing studies. | 7-8 |
|  | 20b | Present results of all statistical syntheses conducted. If meta-analysis was done, present for each the summary estimate and its precision (e.g. confidence/credible interval) and measures of statistical heterogeneity. If comparing groups, describe the direction of the effect. | 7-8 |
|  | 20c | Present results of all investigations of possible causes of heterogeneity among study results. | 8 |
|  | 20d | Present results of all sensitivity analyses conducted to assess the robustness of the synthesized results. | 8 |
| **Reporting biases** | 21 | Present assessments of risk of bias due to missing results (arising from reporting biases) for each synthesis assessed. | 8 |
| **Certainty of evidence** | 22 | Present assessments of certainty (or confidence) in the body of evidence for each outcome assessed. | 8 |
| **DISCUSSION** |  |  |  |
| **Discussion** | 23a | Provide a general interpretation of the results in the context of other evidence. | 9 |
|  | 23b | Discuss any limitations of the evidence included in the review. | 9-13 |
|  | 23c | Discuss any limitations of the review processes used. | 9-13 |
|  | 23d | Discuss implications of the results for practice, policy, and future research. | 14 |
| **OTHER INFORMATION** |  |  |  |
| **Registration and protocol** | 24a | Provide registration information for the review, including register name and registration number, or state that the review was not registered. | 4 |
|  | 24b | Indicate where the review protocol can be accessed, or state that a protocol was not prepared. | 4 |
|  | 24c | Describe and explain any amendments to information provided at registration or in the protocol. | 4 |
| **Support** | 25 | Describe sources of financial or non-financial support for the review, and the role of the funders or sponsors in the review. | 16 |
| **Competing interests** | 26 | Declare any competing interests of review authors. | 16 |
| **Availability of data, code and other materials** | 27 | Report which of the following are publicly available and where they can be found: template data collection forms; data extracted from included studies; data used for all analyses; analytic code; any other materials used in the review. | 16 |

#####

# PRIMSA Abstract Checklist

| **Topic** | **No.** | **Item** | **Reported?** |
| --- | --- | --- | --- |
| **TITLE** |  |  |  |
| **Title** | 1 | Identify the report as a systematic review. | Yes |
| **BACKGROUND** |  |  |  |
| **Objectives** | 2 | Provide an explicit statement of the main objective(s) or question(s) the review addresses. | Yes |
| **METHODS** |  |  |  |
| **Eligibility criteria** | 3 | Specify the inclusion and exclusion criteria for the review. | Yes |
| **Information sources** | 4 | Specify the information sources (e.g. databases, registers) used to identify studies and the date when each was last searched. | Yes |
| **Risk of bias** | 5 | Specify the methods used to assess risk of bias in the included studies. | Yes |
| **Synthesis of results** | 6 | Specify the methods used to present and synthesize results. | Yes |
| **RESULTS** |  |  |  |
| **Included studies** | 7 | Give the total number of included studies and participants and summarise relevant characteristics of studies. | Yes |
| **Synthesis of results** | 8 | Present results for main outcomes, preferably indicating the number of included studies and participants for each. If meta-analysis was done, report the summary estimate and confidence/credible interval. If comparing groups, indicate the direction of the effect (i.e. which group is favoured). | Yes |
| **DISCUSSION** |  |  |  |
| **Limitations of evidence** | 9 | Provide a brief summary of the limitations of the evidence included in the review (e.g. study risk of bias, inconsistency and imprecision). | Yes |
| **Interpretation** | 10 | Provide a general interpretation of the results and important implications. | Yes |
| **OTHER** |  |  |  |
| **Funding** | 11 | Specify the primary source of funding for the review. | Yes |
| **Registration** | 12 | Provide the register name and registration number. | Yes |

*From:* Page MJ, McKenzie JE, Bossuyt PM, Boutron I, Hoffmann TC, Mulrow CD, et al. The PRISMA 2020 statement: an updated guideline for reporting systematic reviews. MetaArXiv. 2020, September 14. DOI: 10.31222/osf.io/v7gm2. For more information, visit: <www.prisma-statement.org>

**Table S2.** The adjusted search terms as per searched electronic databases.

| **PubMed** | | | |
| --- | --- | --- | --- |
|  | #1 | (Monkeypox OR “Monkey Pox” OR “Mpox”) | 3,755 |
|  | #2 | (“Knowledge” OR “ Attitude” OR “Information Sources” OR “Social media” OR “Internet sources” OR “Radio” OR “Television” OR “Newspaper” OR “Friends/Family” OR “WHO website” OR “CDC website” OR “Research articles/ Scientific journals”) | 1,440,822 |
|  | #3 | #1 AND #2 | 254 |
| **Scopus** | | | |
|  | #1 | TITLE-ABS-KEY (Monkeypox OR “Monkey Pox” OR “Mpox”) | 4,345 |
|  | #2 | TITLE-ABS-KEY (“Knowledge” OR “ Attitude” OR “Information Sources” OR “Social media” OR “Internet sources” OR “Radio” OR “Television” OR “Newspaper” OR “Friends/Family” OR “WHO website” OR “CDC website” OR “Research articles/ Scientific journals”) | 4,422,460 |
|  | #3 | #1 AND #2 | 312 |
| **Embase** | | | |
|  | #1 | monkeypox OR 'monkey pox' OR 'mpox' | 4,554 |
|  | #2 | 'knowledge'/exp OR 'knowledge' OR 'attitude'/exp OR 'attitude' OR 'information sources'/exp OR 'information sources' OR 'social media'/exp OR 'social media' OR 'internet sources' OR 'radio'/exp OR 'radio' OR 'television'/exp OR 'television' OR 'newspaper'/exp OR 'newspaper' OR 'friends/family' OR 'who website' OR 'cdc website' OR 'research articles/ scientific journals' | 2,438,035 |
|  | #3 | #1 AND #2 | 395 |
| **Web of Science** | | | |
|  | #1 | ALL=(Monkeypox OR “Monkey Pox” OR “Mpox”) | 4,759 |
|  | #2 | ALL= (“Knowledge” OR “ Attitude” OR “Information Sources” OR “Social media” OR “Internet sources” OR “Radio” OR “Television” OR “Newspaper” OR “Friends/Family” OR “WHO website” OR “CDC website” OR “Research articles/ Scientific journals”) | 3,147,231 |
|  | #3 | #1 AND #2 | 474 |
| **ScienceDirect** | | | |
|  | #1 | (Monkeypox OR “Monkey Pox” OR “Mpox”) | 3,899 |
|  | #2 | (“Knowledge” OR “ Attitude” OR “Information Sources” OR “Social media” OR “Internet sources” OR “Radio” OR “Television” OR “Newspaper” OR “Friends/Family” OR “WHO website” OR “CDC website” OR “Research articles/ Scientific journals”) | 81,360 |
|  | #3 | #1 AND #2 | 398 |

**Table S3**. Quality of the included studies

| Authors | Year | Eligibility criteria | Study subjects and the setting | Exposure measured in a valid and reliable way 'gold standard' | A specified diagnosis or definition | Confounding factors | Dealing with confounding factors | Outcomes measured in a valid and reliable way | Appropriate statistical analysis | Scores (8) | Quality (high, moderate, low) |  |
| --- | --- | --- | --- | --- | --- | --- | --- | --- | --- | --- | --- | --- |
| Abd ElHafeez S, et al. (1) | | 2023 | Yes | Yes | Yes | Yes | Unclear | NA | Yes | Yes | 6 | Moderate |
| Berdida DJE. (2) | | 2023 | Yes | Yes | Yes | Yes | Unclear | NA | Yes | Yes | 6 | Moderate |
| Awoyomi OJ, et al. (3) | | 2023 | Yes | Yes | Yes | Yes | Unclear | NA | Yes | Yes | 6 | Moderate |
| Gonzales-Zamora JA, et al. (4) | | 2023 | Yes | Yes | Yes | Yes | Unclear | NA | Yes | Yes | 6 | Moderate |
| Abu-Farha RK, et al. (5) | | 2023 | Yes | Yes | Yes | Yes | Unclear | NA | Yes | Yes | 6 | Moderate |
| Araoz-Salinas JM, et al. (6) | | 2023 | Yes | Yes | Yes | Yes | Unclear | NA | Yes | Yes | 6 | Moderate |
| Sobaikhi NH, et al. (7) | | 2023 | Yes | Yes | Yes | Yes | Unclear | NA | Yes | Yes | 6 | Moderate |
| Alhasan K, et al. (8) | | 2023 | Yes | Yes | Yes | Yes | Unclear | NA | Yes | Yes | 6 | Moderate |
| Torres TS, et al. (9) | | 2023 | Yes | Yes | Yes | Yes | Unclear | NA | Yes | Yes | 6 | Moderate |
| Shafei AM, et al. (10) | | 2023 | Yes | Yes | Yes | Yes | Unclear | NA | Yes | Yes | 6 | Moderate |
| Al-Mustapha AI, et al. (11) | | 2023 | Yes | Yes | Yes | Yes | Unclear | NA | Yes | Yes | 6 | Moderate |
| Ibrahim AM, et al. (12) | | 2023 | Yes | Yes | Yes | Yes | Unclear | NA | Yes | Yes | 6 | Moderate |
| Fu L, et al. (13) | | 2023 | Yes | Yes | Yes | Yes | Unclear | NA | Yes | Yes | 6 | Moderate |
| Jamaleddine Y, et al. (14) | | 2023 | Yes | Yes | Yes | Yes | Unclear | NA | Yes | Yes | 6 | Moderate |
| Ahmed SK, et al. (15) | | 2023 | Yes | Yes | Yes | Yes | Unclear | NA | Yes | Yes | 6 | Moderate |
| Swed S, et al. (16) | | 2023 | Yes | Yes | Yes | Yes | Unclear | NA | Yes | Yes | 6 | Moderate |
| Elkhwesky Z, et al. (17) | | 2023 | Yes | Yes | Yes | Yes | Unclear | NA | Yes | Yes | 6 | Moderate |
| Youssef D, et al. (18) | | 2023 | Yes | Yes | Yes | Yes | Unclear | NA | Yes | Yes | 6 | Moderate |
| Sahin TK, et al. (19) | | 2022 | Yes | Yes | Yes | Yes | Unclear | NA | Yes | Yes | 6 | Moderate |
| Alshahrani NZ, et al. (20) | | 2022 | Yes | Yes | Yes | Yes | Unclear | NA | Yes | Yes | 6 | Moderate |
| Alshahrani NZ, et al. (21) | | 2022 | Yes | Yes | Yes | Yes | Unclear | NA | Yes | Yes | 6 | Moderate |
| Riad A, et al. (22) | | 2022 | Yes | Yes | Yes | Yes | Unclear | NA | Yes | Yes | 6 | Moderate |
| Kaur A, et al. (23) | | 2022 | Yes | Yes | Yes | Yes | Unclear | NA | Yes | Yes | 6 | Moderate |
| Harapan H, et al. (24) | | 2020 | Yes | Yes | Yes | Yes | Unclear | NA | Yes | Yes | 6 | Moderate |

NA: Not assessed

Aromataris E, Munn Z (Editors). JBI Manual for Evidence Synthesis. JBI, 2020. Available from [https://synthesismanual.jbi.global](https://synthesismanual.jbi.global/).  [https://doi.org/10.46658/JBIMES-20-01](https://jbi-global-wiki.refined.site/space/MANUAL)


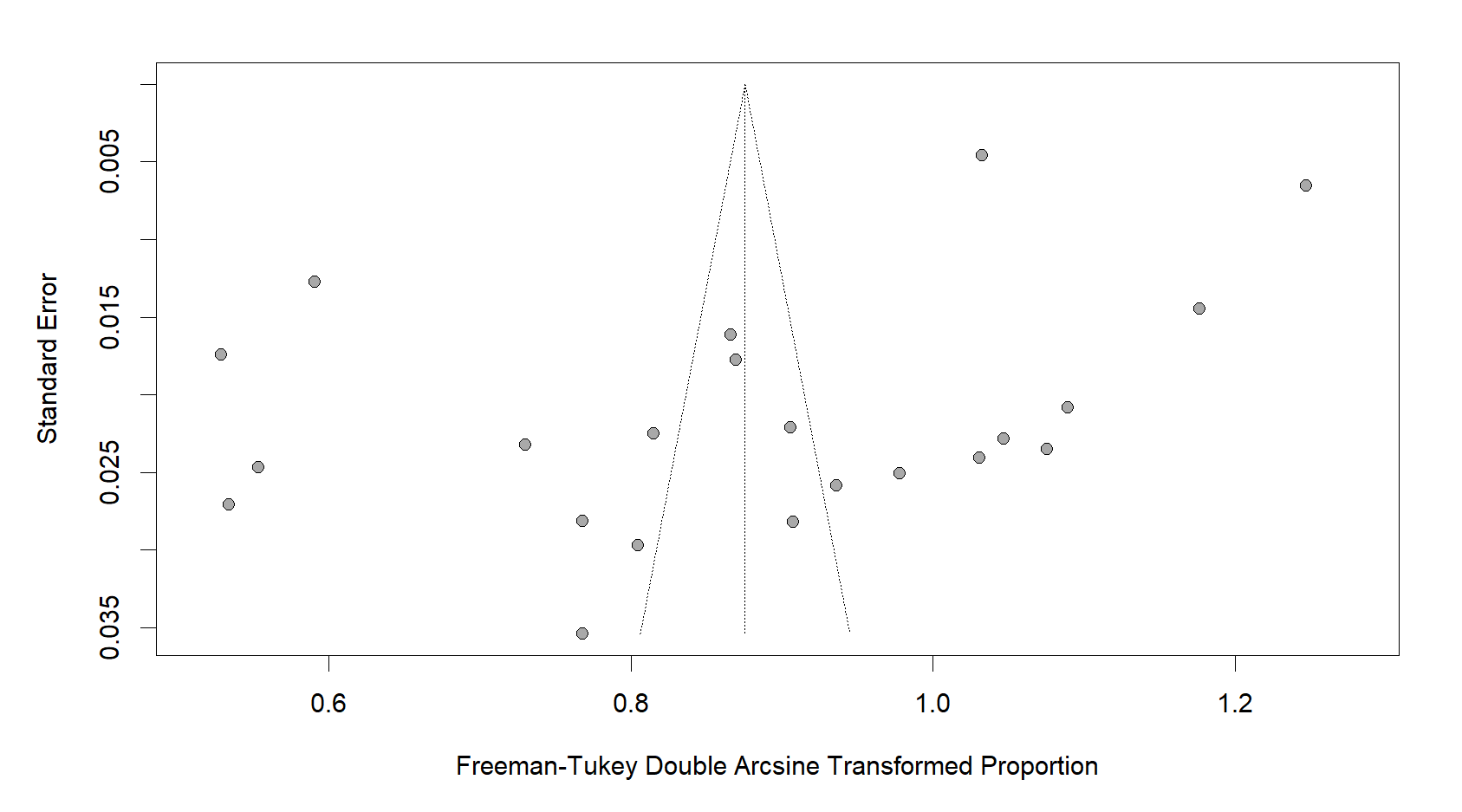


1. Studies evaluating social networks as a source of information about monkeypox.


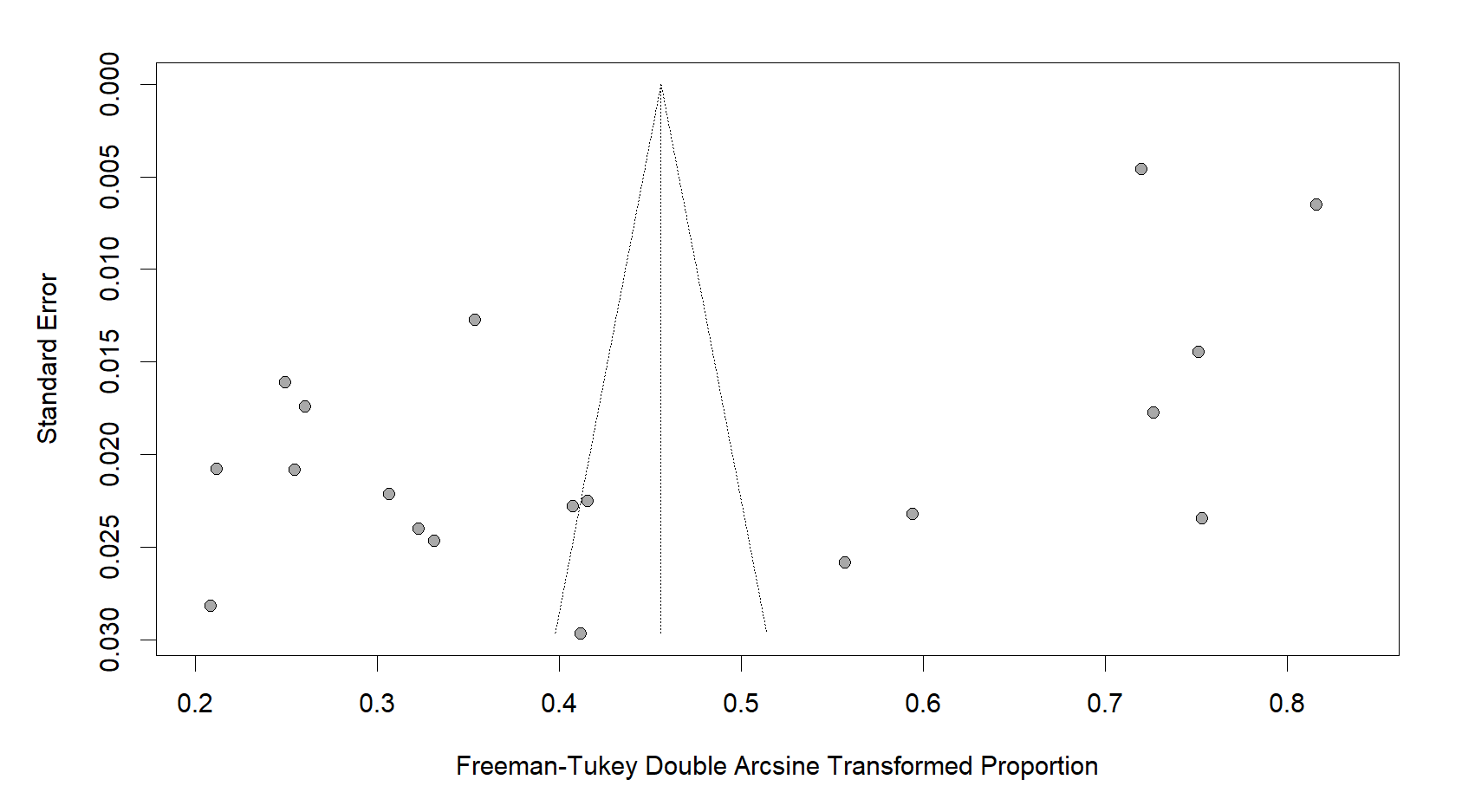


1. Studies evaluate friends or relatives as a source of information about monkeypox.


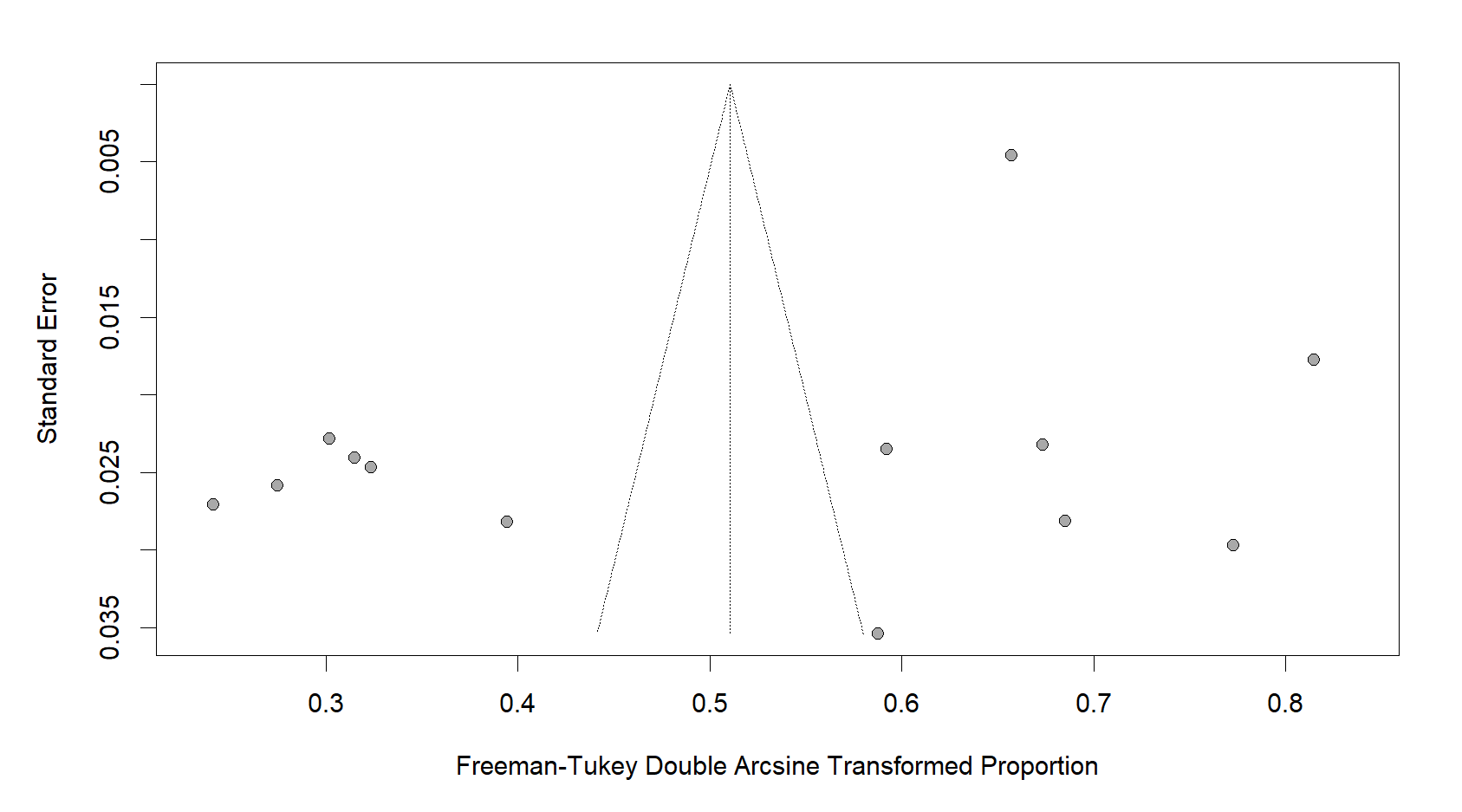


1. Studies evaluating research articles or scientific journals as a source of information on monkeypox.

**Figure S1**: Funnel plot and Egger's test illustrate the publication bias of the included studies.

**
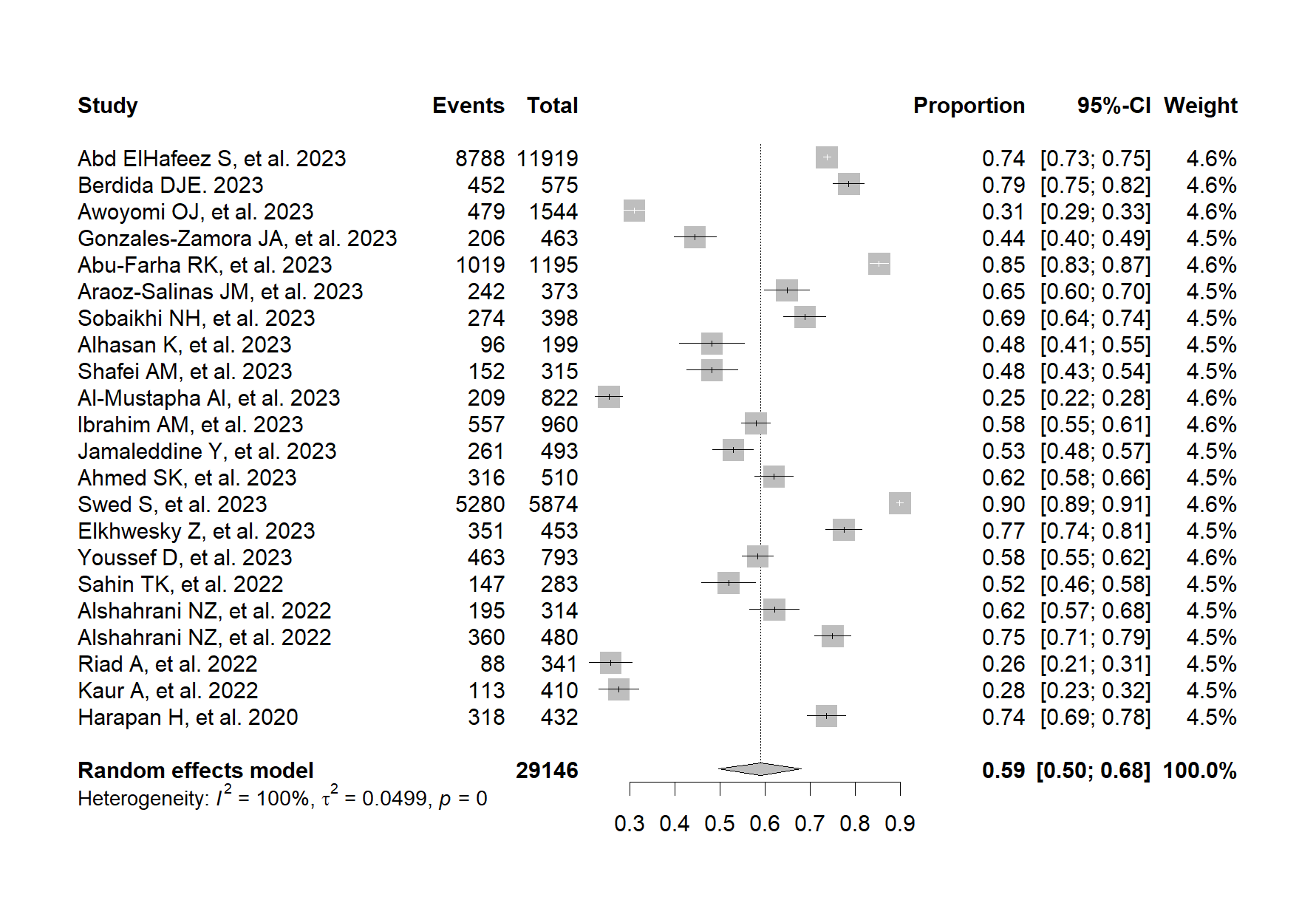
**

**Figure S2.** Forest plot showing the prevalence of social networks as a source of information about monkeypox virus infection.


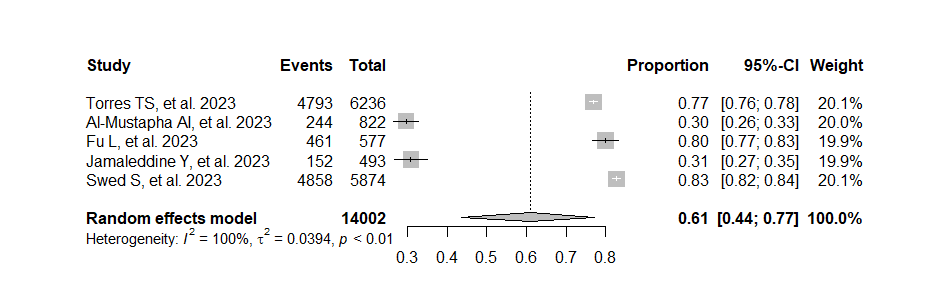


**Figure S3.** Forest plot showing the prevalence of the Internet as a source of information on monkeypox virus infection.

**
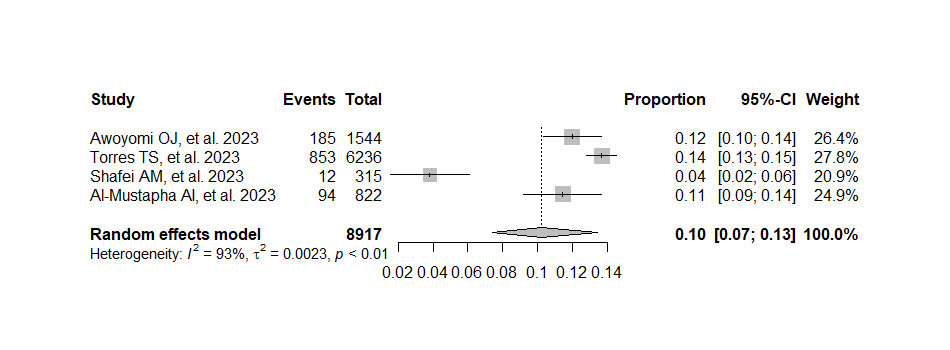
**

**Figure S4.** Forest plot showing the prevalence of Radio as a source of information about monkeypox virus infection.

**
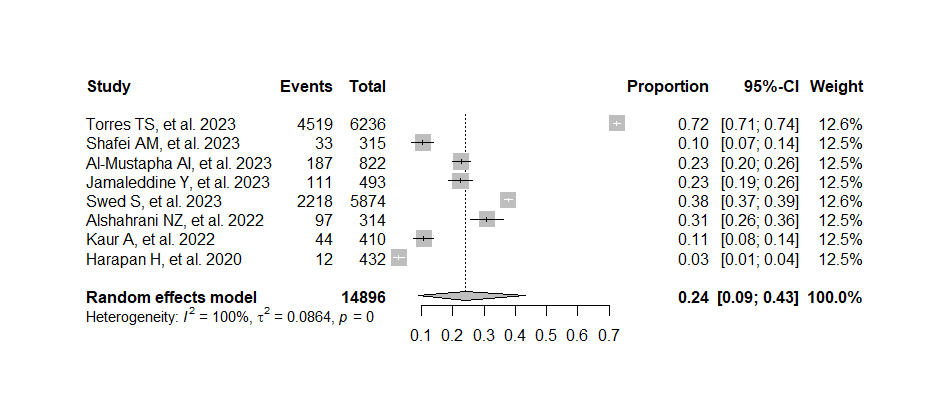
**

**Figure S5.** Forest plot showing the prevalence of Television as a source of information about monkeypox virus infection.

**
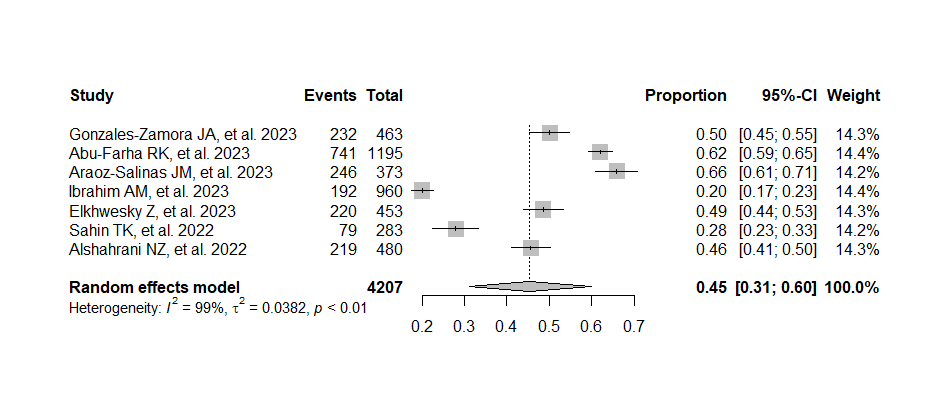
**

**Figure S6.** Forest plot showing the prevalence of Radio/Television as a source of information about monkeypox virus infection.

**
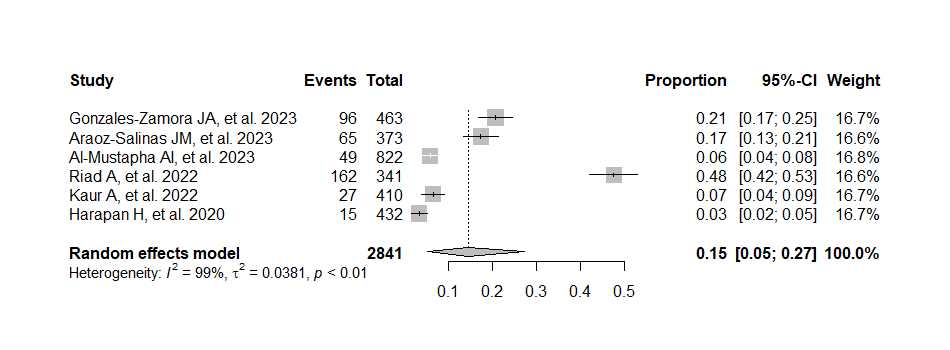
**

**Figure S7.** Forest plot showing the prevalence of newspaper as a source of information on monkeypox virus infection.

**
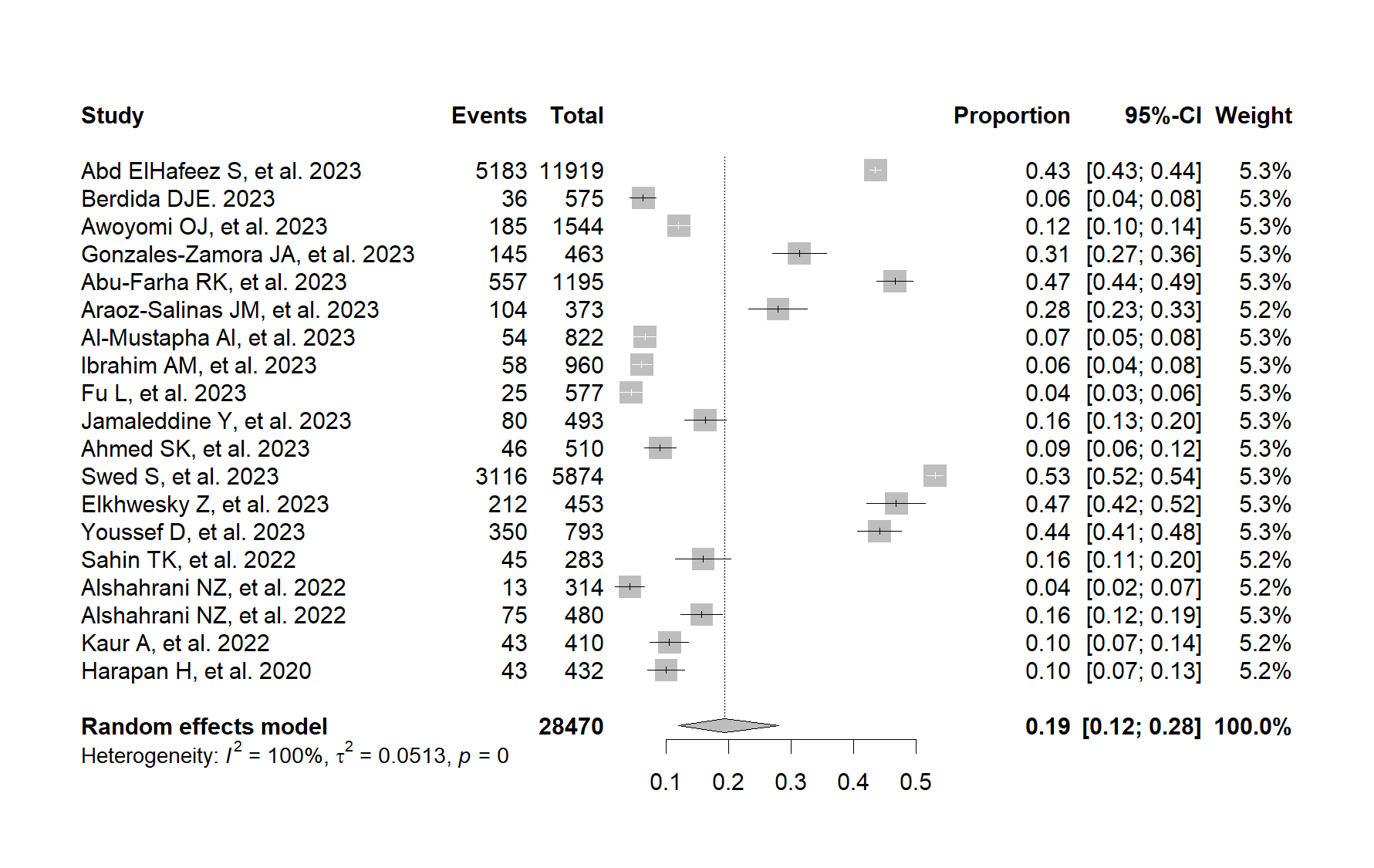
**

**Figure S8.** Forest plot showing the prevalence of Friends/Family as a source of information about monkeypox virus infection.

**
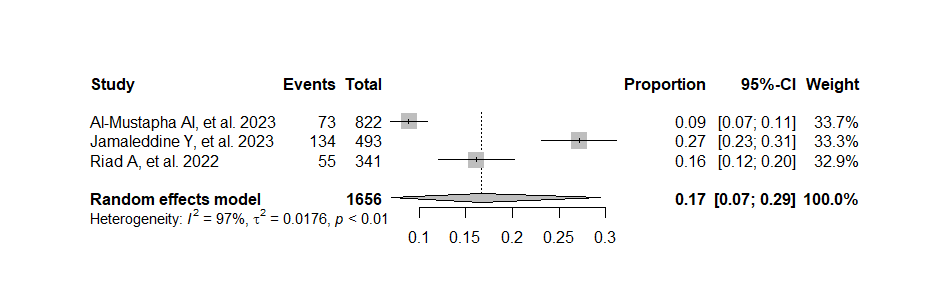
**

**Figure S9.** Forest plot showing the prevalence of the WHO website as a source of information on monkeypox virus infection.

**
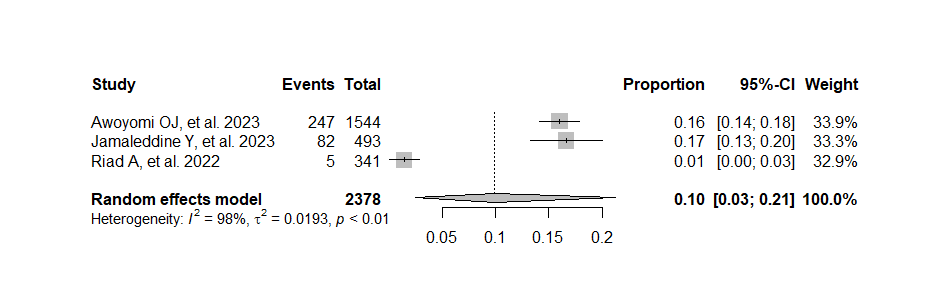
**

**Figure S10.** Forest plot showing the prevalence of the CDC website as a source of information on monkeypox virus infection.

**
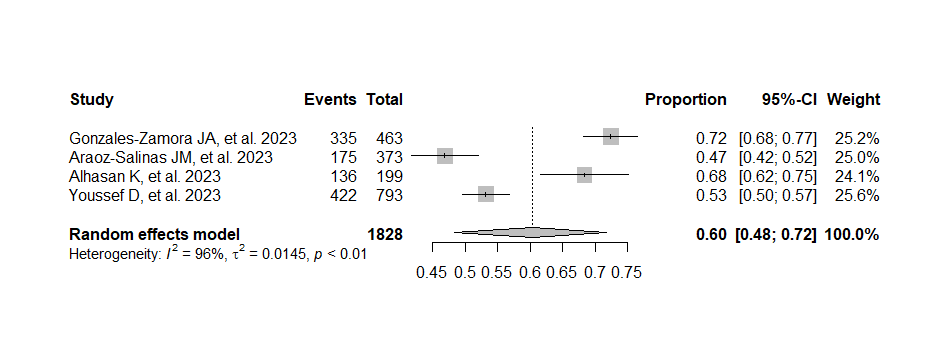
**

**Figure S11.** Forest plot showing the prevalence of WHO and CDC websites as a source of information on monkeypox virus infection.

**
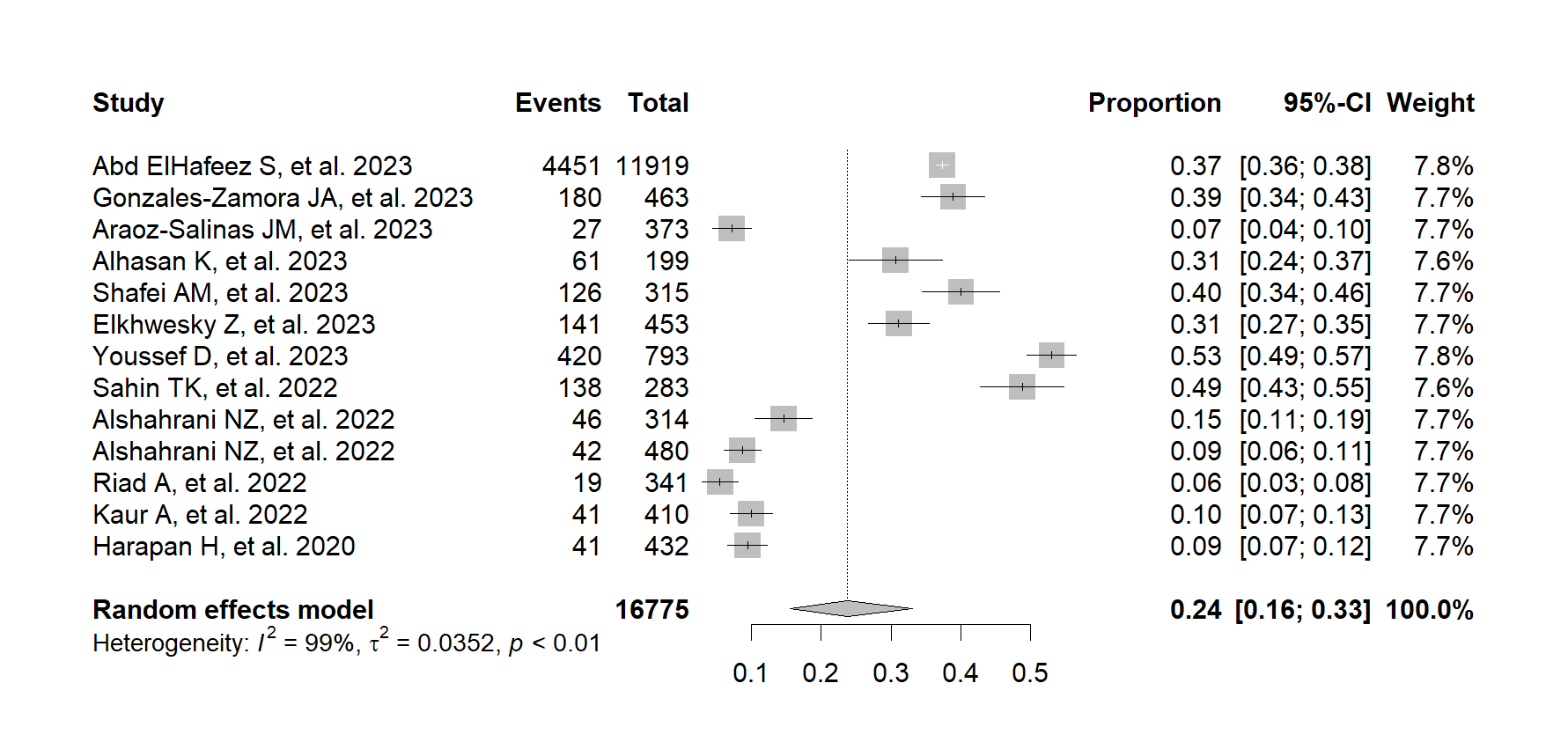
**

**Figure S12.** Forest plot showing the prevalence of research articles/scientific journals as a source of information on monkeypox virus infection.


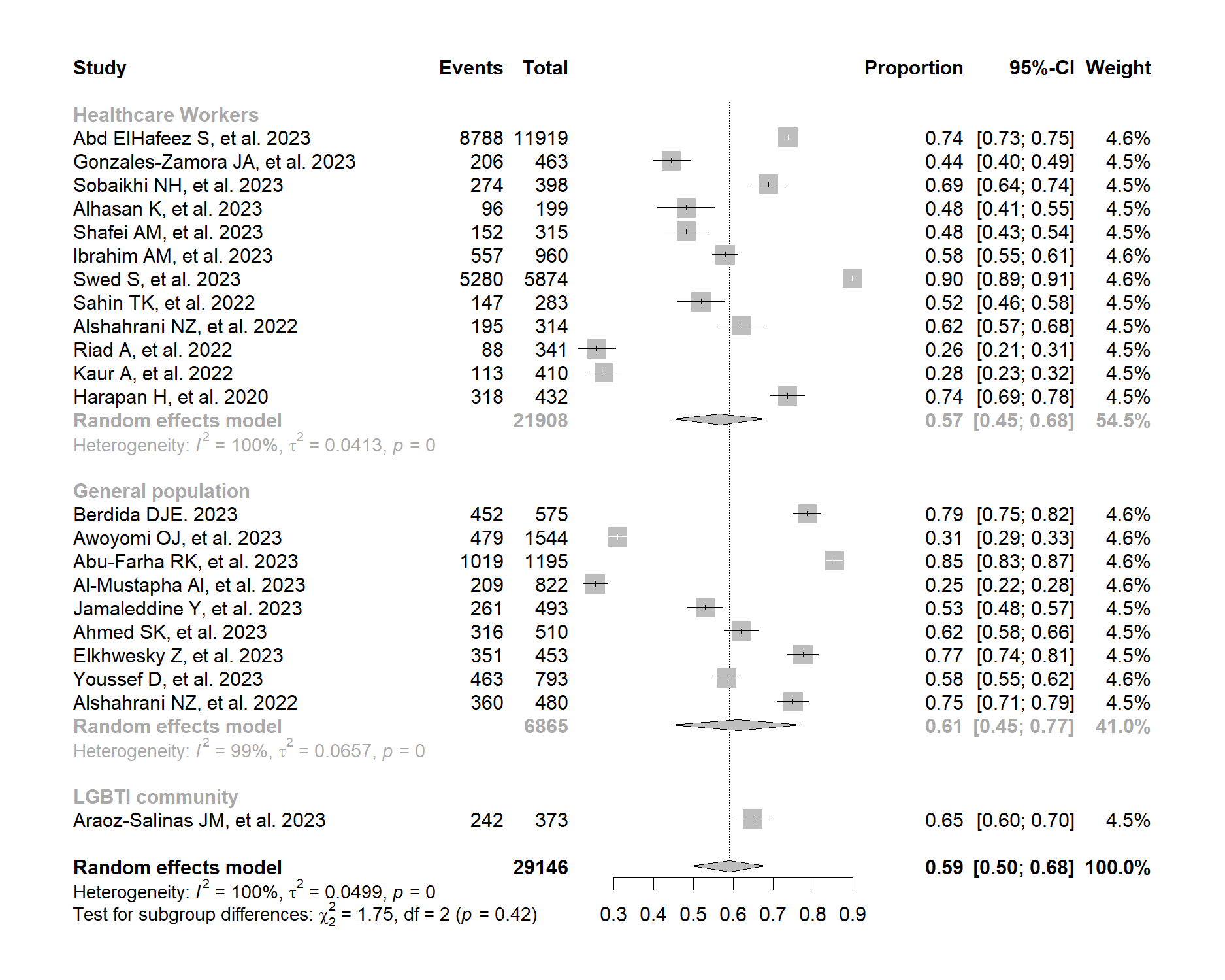


**Figure S13.** Graphical representation of the prevalence of information sources in social networks about monkeypox virus among study participants.


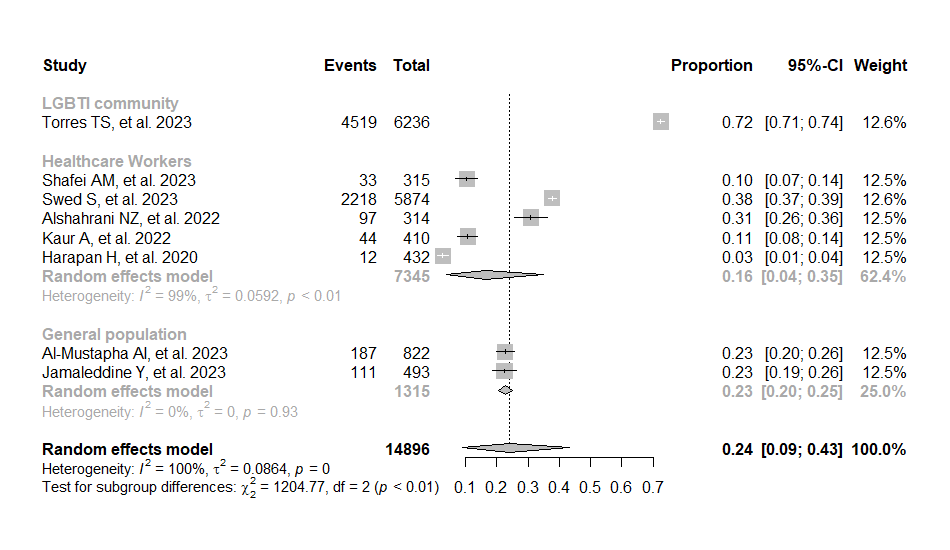


**Figure S14.** Graphical representation of the prevalence of information sources in television about monkeypox virus among study participants.


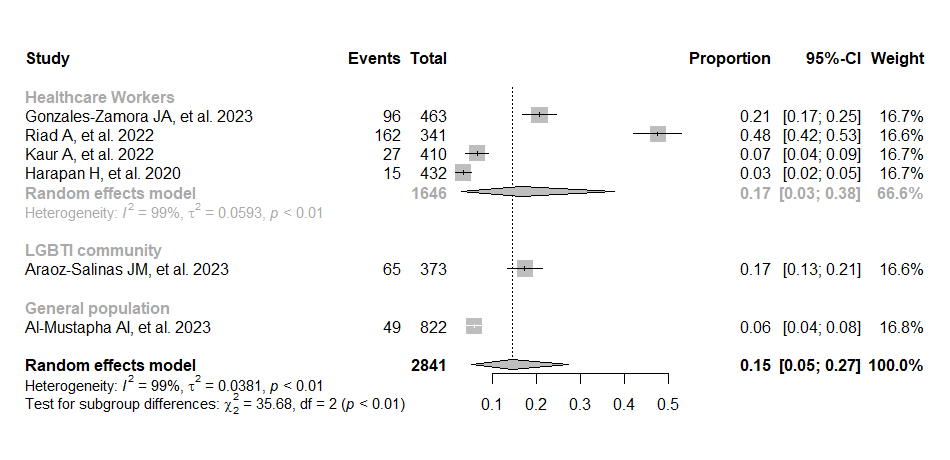


**Figure S15.** Graphical representation of the prevalence of information sources in newspapers about monkeypox virus among study participants.


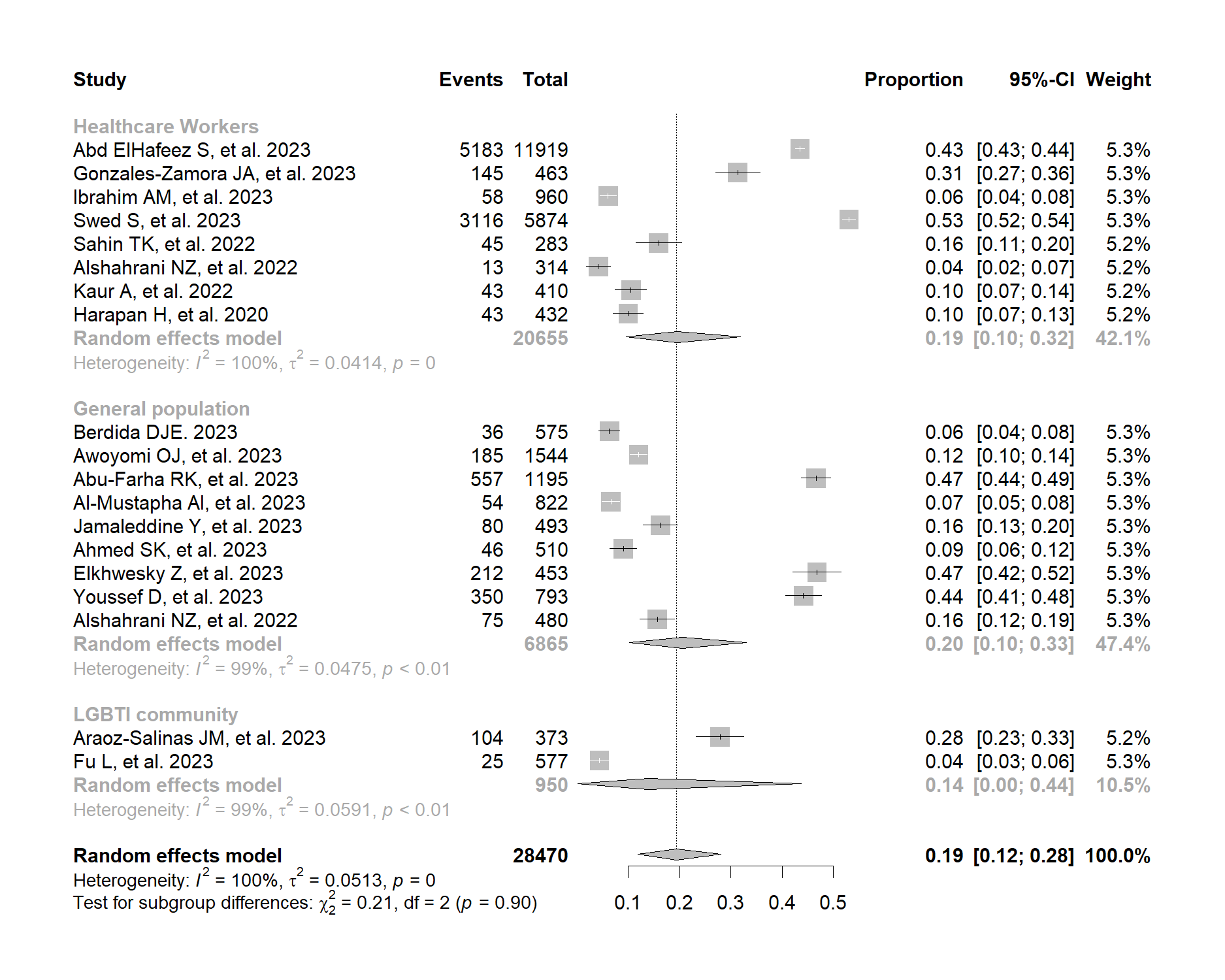


**Figure S16.** Graphical representation of the prevalence of information sources in Friends/Family about monkeypox virus among study participants.


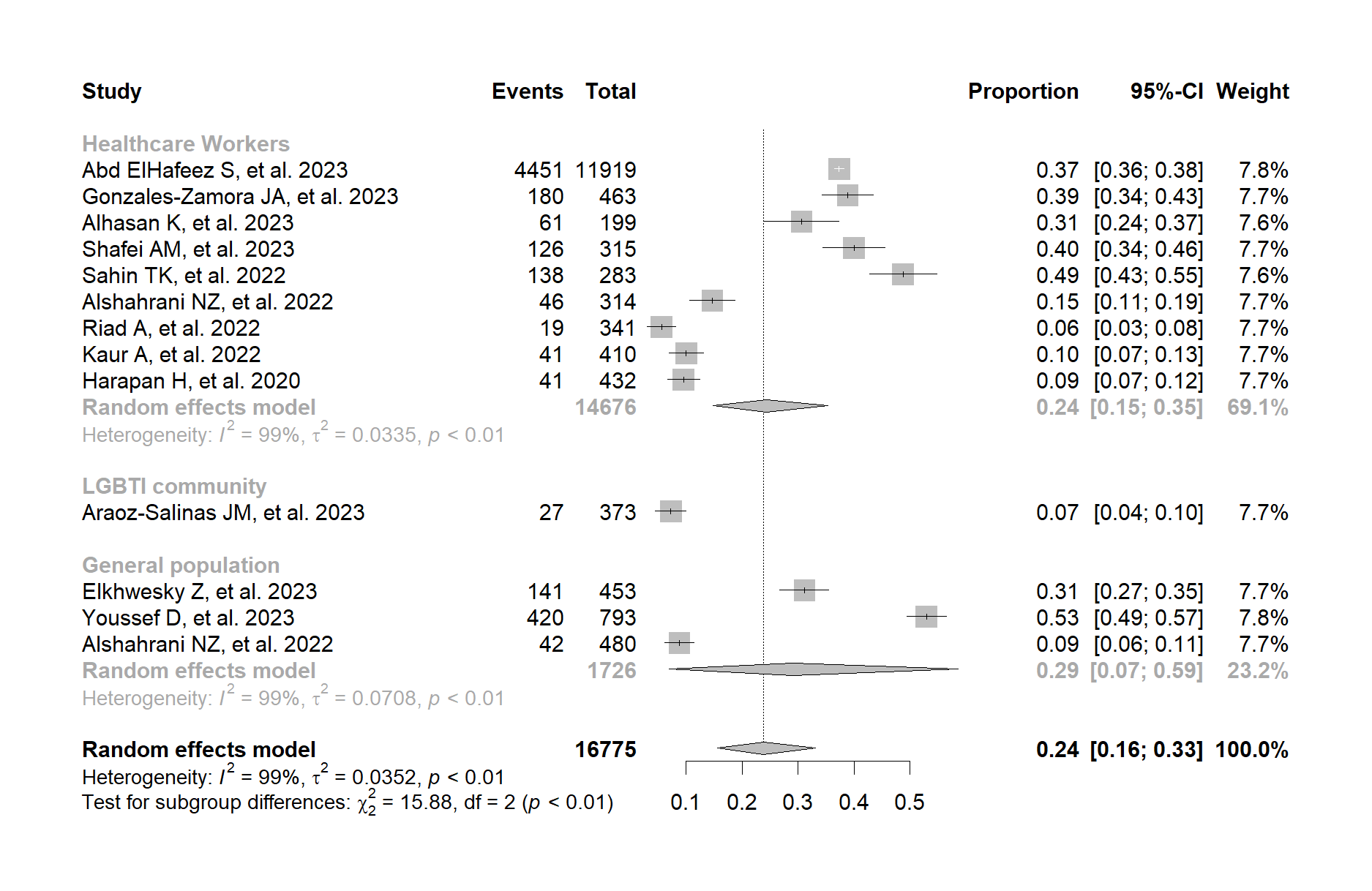


**Figure S17.** Graphical representation of the prevalence of information sources in Research articles/ Scientific journals about monkeypox virus among study participants.

References

1. Abd ElHafeez S, Gebreal A, Khalil MA, Youssef N, Sallam M, Elshabrawy A, et al. Assessing disparities in medical students’ knowledge and attitude about monkeypox: a cross-sectional study of 27 countries across three continents. Front Public Health. 2023;11:1192542.

2. Berdida DJE. Population-based survey of human monkeypox disease knowledge in the Philippines: An online cross-sectional study. J Adv Nurs. julio de 2023;79(7):2684-94.

3. Awoyomi OJ, Njoga EO, Jaja IF, Oyeleye FA, Awoyomi PO, Ibrahim MA, et al. Mpox in Nigeria: Perceptions and knowledge of the disease among critical stakeholders-Global public health consequences. PloS One. 2023;18(3):e0283571.

4. Gonzales-Zamora JA, Soriano-Moreno DR, Soriano-Moreno AN, Ponce-Rosas L, Sangster-Carrasco L, De-Los-Rios-Pinto A, et al. Level of Knowledge Regarding Mpox among Peruvian Physicians during the 2022 Outbreak: A Cross-Sectional Study. Vaccines. 12 de enero de 2023;11(1):167.

5. Abu-Farha RK, Alzoubi KH, Mukattash TL, Alkhawaldeh R, Barakat M, Thiab S. Public Knowledge and Perceptions about the Emerging Human Mpox in Jordan: A Cross-Sectional Study. Trop Med Infect Dis. 5 de enero de 2023;8(1):41.

6. Araoz-Salinas JM, Ortiz-Saavedra B, Ponce-Rosas L, Soriano-Moreno DR, Soriano-Moreno AN, Alave J, et al. Perceptions and Intention to Get Vaccinated against Mpox among the LGBTIQ+ Community during the 2022 Outbreak: A Cross-Sectional Study in Peru. Vaccines. 21 de mayo de 2023;11(5):1008.

7. Sobaikhi NH, Alshahrani NZ, Hazazi RS, Al-Musawa HI, Jarram RE, Alabah AE, et al. Health Workers’ Knowledge and Attitude towards Monkeypox in Southwestern Saudi Arabia: A Cross-Sectional Study. Diseases. junio de 2023;11(2):81.

8. Alhasan K, Sallam M, Aljamaan F, Ali T, Al-Jedai A, Nazmi A, et al. Mpox Perceptions and Vaccine Advocacy among the Healthcare Workers of Solid Organ Transplant Centers: A Multicenter, Cross-Sectional Survey in Saudi Arabia. Healthc Basel Switz. 17 de febrero de 2023;11(4):603.

9. Torres TS, Silva MST, Coutinho C, Hoagland B, Jalil EM, Cardoso SW, et al. Evaluation of Mpox Knowledge, Stigma, and Willingness to Vaccinate for Mpox: Cross-Sectional Web-Based Survey Among Sexual and Gender Minorities. JMIR Public Health Surveill. 17 de julio de 2023;9:e46489.

10. Shafei AM, Al-Mosaa KM, Alshahrani NZ, ALAmmari MHM, Almuhlafi MOO, Draim NHAA, et al. Resident Physicians’ Knowledge and Preparedness Regarding Human Monkeypox: A Cross-Sectional Study from Saudi Arabia. Pathog Basel Switz. 26 de junio de 2023;12(7):872.

11. Al-Mustapha AI, Ogundijo OA, Sikiru NA, Kolawole B, Oyewo M, El-Nadi H, et al. A cross-sectional survey of public knowledge of the monkeypox disease in Nigeria. BMC Public Health. 29 de marzo de 2023;23(1):591.

12. Ibrahim AM, Zaghamir DEF. Knowledge and attitudes towards mpox and effect of intervention among College of Applied Medical Sciences students. Libyan J Med. diciembre de 2023;18(1):2222448.

13. Fu L, Sun Y, Li Y, Wang B, Yang L, Tian T, et al. Perception of and Vaccine Readiness towards Mpox among Men Who Have Sex with Men Living with HIV in China: A Cross-Sectional Study. Vaccines. 23 de febrero de 2023;11(3):528.

14. Jamaleddine Y, El Ezz AA, Mahmoud M, Ismail O, Saifan A, Mayta Z, et al. Knowledge and attitude towards monkeypox among the Lebanese population and their attitude towards vaccination. J Prev Med Hyg. marzo de 2023;64(1):E13-26.

15. Ahmed SK, Abdulqadir SO, Omar RM, Abdullah AJ, Rahman HA, Hussein SH, et al. Knowledge, Attitude and Worry in the Kurdistan Region of Iraq during the Mpox (Monkeypox) Outbreak in 2022: An Online Cross-Sectional Study. Vaccines. 8 de marzo de 2023;11(3):610.

16. Swed S, Bohsas H, Patwary MM, Alibrahim H, Rakab A, Nashwan AJ, et al. Knowledge of mpox and its determinants among the healthcare personnel in Arabic regions: A multi-country cross-sectional study. New Microbes New Infect. 1 de septiembre de 2023;54:101146.

17. Elkhwesky Z, Derhab N, Elkhwesky FFY, Abuelhassan AE, Hassan H. Hotel employees’ knowledge of monkeypox’s source, symptoms, transmission, prevention, and treatment in Egypt. Travel Med Infect Dis. 2023;53:102574.

18. Youssef D, Abboud E, Kawtharani M, Zheim Z, Abou Arrage N, Youssef J. When a neglected tropical zoonotic disease emerges in non-endemic countries: need to proactively fill the unveiled knowledge gaps towards human monkeypox among the Lebanese population. J Pharm Policy Pract. 7 de marzo de 2023;16(1):39.

19. Sahin TK, Erul E, Aksun MS, Sonmezer MC, Unal S, Akova M. Knowledge and Attitudes of Turkish Physicians towards Human Monkeypox Disease and Related Vaccination: A Cross-Sectional Study. Vaccines. 21 de diciembre de 2022;11(1):19.

20. Alshahrani NZ, Mitra S, Alkuwaiti AA, Alhumam MN, Altmimi SMB, Alamri MHM, et al. Medical Students’ Perception Regarding the Re-emerging Monkeypox Virus: An Institution-Based Cross-Sectional Study From Saudi Arabia. Cureus. agosto de 2022;14(8):e28060.

21. Alshahrani NZ, Alzahrani F, Alarifi AM, Algethami MR, Alhumam MN, Ayied HAM, et al. Assessment of Knowledge of Monkeypox Viral Infection among the General Population in Saudi Arabia. Pathog Basel Switz. 11 de agosto de 2022;11(8):904.

22. Riad A, Drobov A, Rozmarinová J, Drapáčová P, Klugarová J, Dušek L, et al. Monkeypox Knowledge and Vaccine Hesitancy of Czech Healthcare Workers: A Health Belief Model (HBM)-Based Study. Vaccines. 26 de noviembre de 2022;10(12):2022.

23. Kaur A, Goel R, Singh R, Bhardwaj A, Kumari R, Gambhir RS. Identifying monkeypox: do dental professionals have adequate knowledge and awareness? Rocz Panstw Zakl Hig. 2022;73(3):365-71.

24. Harapan H, Setiawan AM, Yufika A, Anwar S, Wahyuni S, Asrizal FW, et al. Knowledge of human monkeypox viral infection among general practitioners: a cross-sectional study in Indonesia. Pathog Glob Health. marzo de 2020;114(2):68-75.
